# Assessing the variability in experimental hut trials evaluating insecticide-treated nets against malaria vectors

**DOI:** 10.1016/j.crpvbd.2023.100115

**Published:** 2023-02-09

**Authors:** Joseph D. Challenger, Rebecca K. Nash, Corine Ngufor, Antoine Sanou, K. Hyacinthe Toé, Sarah Moore, Patrick K. Tungu, Mark Rowland, Geraldine M. Foster, Raphael N’Guessan, Ellie Sherrard-Smith, Thomas S. Churcher

**Affiliations:** a Medical Research Council Centre for Global Infections Disease Analysis, Department of Infectious Disease Epidemiology, Imperial College London, London, UK; b Centre de Recherches Entomologiques de Cotonou, Cotonou, Benin; c London School of Hygiene and Tropical Medicine, London, UK; d Centre National de Recherche et de Formation sur le Paludisme, Burkina Faso; e Vector Control Product Testing Unit (VCPTU) Ifakara Health Institute, Environmental Health, and Ecological Sciences, Bagamoyo, Tanzania; f Vector Biology Unit, Department of Epidemiology and Public Health, Swiss Tropical & Public Health Institute, Basel, Switzerland; g Faculty of Science, University of Basel, Basel, Switzerland; h The Nelson Mandela African Institution of Science and Technology (NM-AIST), Tengeru, Arusha, Tanzania; i National Institute for Medical Research, Amani Medical Research Centre, Muheza, Tanzania; j Vector Biology Department, Liverpool School of Tropical Medicine, Liverpool, UK; k Institut Pierre Richet, Bouaké, Republic of Côte d’Ivoire

**Keywords:** Experimental hut trials, Insecticide-treated nets, Long-lasting insecticidal nets, Vector control, *Anopheles*, Power analysis

## Abstract

Experimental hut trials (EHTs) are used to evaluate indoor vector control interventions against malaria vectors in a controlled setting. The level of variability present in the assay will influence whether a given study is well powered to answer the research question being considered. We utilised disaggregated data from 15 previous EHTs to gain insight into the behaviour typically observed. Using simulations from generalised linear mixed models to obtain power estimates for EHTs, we show how factors such as the number of mosquitoes entering the huts each night and the magnitude of included random effects can influence study power. A wide variation in behaviour is observed in both the mean number of mosquitoes collected per hut per night (ranging from 1.6 to 32.5) and overdispersion in mosquito mortality. This variability in mortality is substantially greater than would be expected by chance and should be included in all statistical analyses to prevent false precision of results. We utilise both superiority and non-inferiority trials to illustrate our methodology, using mosquito mortality as the outcome of interest. The framework allows the measurement error of the assay to be reliably assessed and enables the identification of outlier results which could warrant further investigation. EHTs are increasingly playing an important role in the evaluation and regulation of indoor vector control interventions so it is important to ensure that these studies are adequately powered.

## Highlights

• Insecticide-treated products are a vital tool in the fight against malaria.
• Experimental hut trials can be used to evaluate products designed for indoor use.
• We use simulation-based methods to provide power estimates for hut trials.
• Data from previous hut trials help us understand the amount of variability in the assay.
• Realistic case studies are used, to show power estimates for a range of scenarios.

## Graphical abstract

**Figure:**
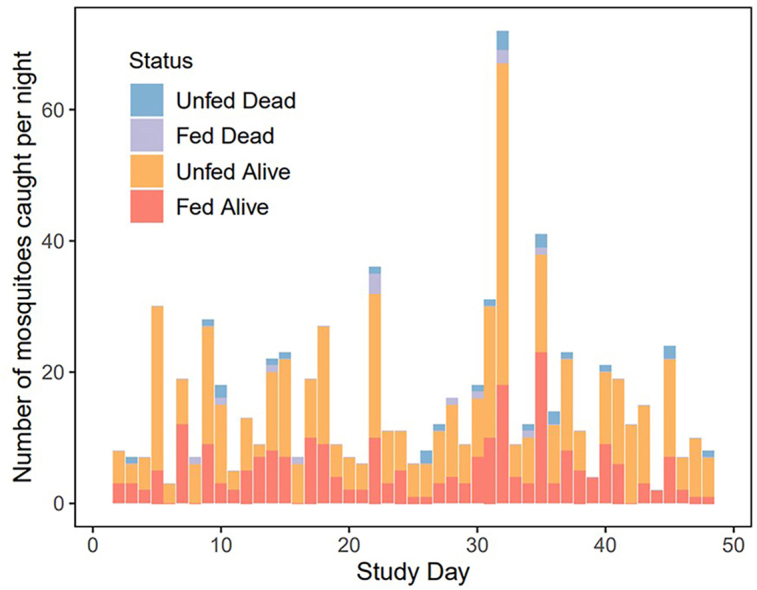

## 1 Introduction

The widespread distribution of insecticide-treated nets (ITNs) across sub-Saharan Africa was a major driver in the reduction of malaria incidence observed between 2000 and 2015 (Bhatt et al., 2015). ITNs remain a valuable tool against malaria in settings in which most mosquitoes seek to feed at night, where they are an integral part of malaria control programmes. In Africa the rise in mosquitoes resistant to pyrethroids, the class of insecticide currently used on all nets, is thought to have diminished the impact of ITN distribution (Protopopoff et al., 2018; Staedke et al., 2020). Fully functioning ITNs will kill mosquitoes, thereby reducing the size of the local mosquito population and generating a community-level benefit (Hawley et al., 2003), as well as providing a protective barrier to individuals sleeping under them.

The selection for and spread of pyrethroid-resistant mosquitoes mean there is a need for alternative classes of insecticides that are both safe for humans to sleep under and effective at killing *Anopheles* spp*.* mosquitoes. New ITN products are assessed for their safety and entomological efficacy by the World Health Organisation (WHO) before they can be recommended for purchase by international donors such as the Global Fund (WHO, 2020). Novel types of ITN that have a mode of action that is substantially different from those currently in use must also demonstrate epidemiological impact which is typically done using cluster randomised control trials (CRCTs) (WHO, 2017).

The entomological efficacy of ITNs is typically assessed in experimental hut trials (EHTs) which provide an opportunity to evaluate indoor vector control interventions in a controlled setting that recreates the way in which products are used as a public health tool. In these studies, volunteers sleep inside specially designed huts: mosquitoes can enter but are prevented from escaping by the presence of exit traps. In this way, the proportion of mosquitoes that are killed, or that successfully blood feed, can be contrasted across different interventions, e.g. volunteers sleeping under an ITN *vs* an untreated net. Different hut designs have been used for evaluating insecticide-treated products and their design can influence results (Massue et al., 2016; Oumbouke et al., 2017). EHTs can be used to evaluate ITNs against laboratory-reared mosquitoes or wild, free-flying mosquitoes that enter naturally. These studies are a relatively quick and economical method of evaluating different ITNs in real-world settings. EHTs data are currently being used to support WHO policy on the deployment of new types of indoor residual spraying products. It has also been suggested that novel ITNs with a fast, neuro-acting insecticides may not need to demonstrate epidemiological evidence of impact but could be recommended for use on EHT data alone (Sherrard-Smith et al., 2022a). The WHO have provided clear instructions on how EHTs should be conducted, outlining the arms that should be included, how interventions/volunteers should be rotated, blinding, replication and how data should be analysed (WHO, 2013). Nevertheless, there is relatively little information on how large studies should be, and formal power analyses are rarely conducted. With their increasing use in policy and product development, it is important to understand how robust the complex biological assay is, to allow studies to be suitably powered to answer the research or regulatory questions posed.

EHT data have been used to understand the ability of control interventions to induce mosquito mortality and inhibit blood-feeding. A number of different trial designs may be used, but the two most widely utilised are superiority trials and non-inferiority trials. A superiority trial could be performed to demonstrate that a particular insecticide-treated product is superior to another product (for example, a net not treated with insecticide, or a pyrethroid-only ITN). Non-inferiority trials are suitable for demonstrating that a novel product is not less effective than the currently used product, at least by no more than a predetermined margin (the non-inferiority margin) (Schumi & Wittes, 2011). It has been proposed that new ITN products that are in the same product class to existing ITNs that have demonstrated epidemiological impact, could be evaluated by conducting non-inferiority trials (WHO, 2021).

Data from EHTs are typically analysed using logistic regression models. Since a study typically will include multiple huts, these models should account for variation that can arise due to differences between the huts and proximity to mosquito breeding sites. In some trials volunteers who sleep within each hut are also rotated nightly between huts as individuals vary in their attractiveness to mosquitoes due to differences in their mass and body odour (Takken & Verhulst, 2013). Additional terms, which we here incorporate as random effects, are included in the model to account for differences between huts and volunteers. This makes the assessment of a trial’s statistical power more complicated. To properly assess the study power, it is possible to simulate many EHTs *in silico* and perform an analysis of the data generated for each simulated trial, as outlined in Johnson et al. (2015). In this way, we can explore how different factors in a study, such as the number of mosquitoes entering each hut, will influence a trial’s statistical power. In addition, this is a complex biological assay, and it is likely that there are other sources of variability in results that are not accounted for by differences between huts or volunteers. Failure to capture this additional uncertainty risks designing underpowered studies and generating overly precise results which could be misleading.

This paper is structured as follows. In the *Methods* section, we outline a typical design for an EHT and introduce the regression framework that is used to analyse these data. We show how the output from the regression model should be used, firstly for a superiority trial, then for a non-inferiority trial. We show how the non-inferiority assessment is made, using output from the regression model to construct an odds ratio for the mortality (or blood-feeding inhibition) induced by the two ITNs. We then outline how the statistical power of a given trial is determined. Though methods for determining statistical power have been available for a while (Warton & Hui, 2011; Johnson et al., 2015) they are seldomly reported, and it is often unclear whether they have been conducted. This could be in part because it is unclear how these power calculations should be parameterised. In the *Results* section, we summarise data from previous EHTs to gain insight into the dynamics typically observed in these trials and sources of variability. Informed by these data, we explore the factors that influence the statistical power of an EHT and suggest ways to ensure that future studies are sufficiently powered. The *Tutorial* in the Supplementary file 1 accompanying this article allows the reader to gain an understanding of the methods used in this work.

## 2 Methods

### 2.1 Illustrative trial design

Here we define an illustrative EHT design which we use throughout to show how a variety of factors influence study power and results. Results will vary if different trial designs are used. We shall consider an EHT with seven arms, which requires seven experimental huts and seven volunteers to sleep under the nets each night. An untreated net is used as the control arm, and six treatment ITN arms are included. In this example, the treatment arms are: the investigational item unwashed and washed 20 times (as a proxy for ageing), and two positive control ITNs, each of which are unwashed and washed 20 times. These ITNs could contain different insecticides or combinations of insecticides. Due to the fact that ITN efficacy needs to last for an extended period of time (typically in malaria-endemic settings, new ITNs are distributed every three years), the durability of the product can be an important part of the evaluation. In these studies, ITNs are usually washed 20 times to approximate the effects of 3 years of ageing (WHO, 2013) although, alternatively, field-aged nets may also be used.

Over the course of the trial, the nets and sleepers are rotated around the 7 huts in a Latin square design (LSD). In this illustrative EHT, each net remains in a particular hut for 7 nights: note that some trials use 6 nights. In this EHT, one rotation of the LSD generates 7^3^ (343) data points. Rest days are included in the trial design, during which huts are aired for 24 ​h to minimise any carry over effects, before a new net is introduced into each hut. Therefore, a full rotation of the LSD for a 7-arm trial requires 56 days to complete.

Both the mosquito mortality and blood-feeding inhibition induced by the ITNs can be estimated using logistic regression modelling. In this work we will consider mosquito mortality, but the analysis for blood-feeding inhibition is very similar. The total number of mosquitoes that enter each hut each night is recorded as well as the number of mosquitoes killed. As the lethality induced by the ITN may be delayed, the mortality assessment is usually made after the mosquitoes have been monitored for a period of time, which depends on the mode of action of the insecticide. A period of 24 ​h is used as a default, although a period of at least 72 ​h is used for ITNs with a delayed mode of action such as pyrrole insecticides.

For the modelling, let *N* represent the total number of mosquitoes that enter a hut on a given night. We use the subscripts *j* and *k* to denote that a data point corresponds to hut *j* and volunteer *k*. Each observation is identified by subscript *i* which takes a unique value for each data point (hence in this case *i* = (1, 2, …, 343). We assume that the number of dead mosquitoes recorded, $r_{ijk}$, is binomially distributed i.e. $r_{ijk}∼B\left(N_{ijk},p_{ijk}\right)$. The regression model describes the mean mosquito mortality, $p_{ijk}$, in terms of its log-odds transformed value. It contains fixed effects to account for the ITN in use, and random effects to account for variations in the mortality observed due to other factors. We allow for variation in mosquito mortality due to the hut and volunteer in question. We also allow for any unexplained variation observed in the proportion of mosquitoes killed, by including an observation-level random effect (Warton and Hui, 2011; Johnson et al., 2015). This is included to capture any additional variability (i.e. overdispersion in mosquito mortality) not explained by the net type, hut, volunteer or the binomial distribution. As outlined in the *Tutorial* (Supplementary file 1), a Likelihood Ratio Test can be used to check whether the inclusion of this term is justified, in terms of the improvement in model fit. We write the overall model for the mortality (for data point *i*) as:

(1) $$\log\left(\frac{p_{ijk}}{1-p_{ijk}}\right)=\beta_{0}+\sum_{m=1}^{6}\beta_{m}x_{im}+b_{i}+h_{j}+v_{k}\text{.}$$

Here $x_{im}$ is an indicator variable, denoting which of the nets is used (and whether or not it has been washed, if both washed and unwashed ITNs are included in the trial) for data point *i*, and $\beta_{m}$ is the difference in the mortality induced (on the log-odds scale) by net m, relative to that induced by the control net ($\beta_{0}$). This model contains three random effects for the hut used ($h_{j}$), the volunteer sleeping under the net ($v_{k}$), and an observation-level random effect ($b_{i}$). All random effects are assumed to be normally distributed:

$$h_{j}∼N\left(0,\sigma_{h}\right),j=1,2,\ldots ,7$$

$$v_{k}∼N\left(0,\sigma_{v}\right),k=1,2,\ldots ,7$$

(2) $$b_{i}∼N\left(0,\sigma_{b}\right),i=1,2,...,343$$

**Fig. 1:**
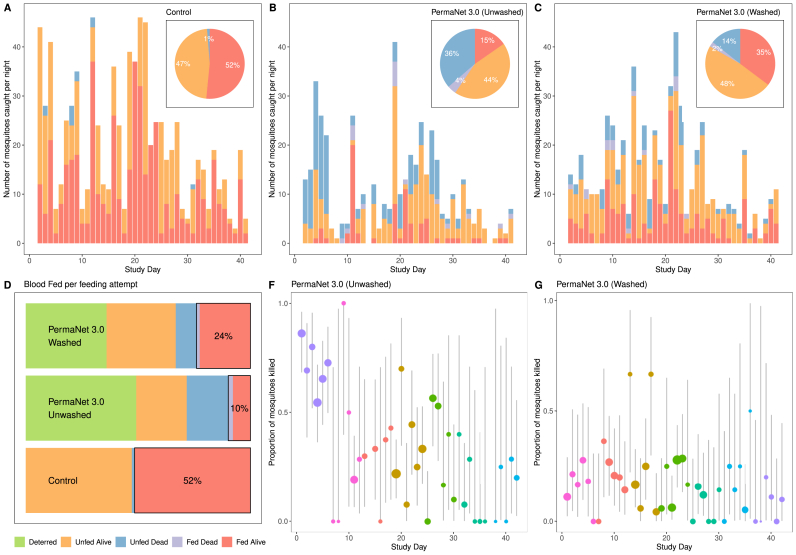
Data from selected arms of an experimental hut trial, showing nightly variability in mosquito mortality, blood-feeding, and numbers caught. **A** Breakdown of mosquito numbers in the control arm (untreated net) over the course of the trial. The height of each bar indicates the total number of mosquitoes entering the hut each night. Bar colour denotes the mosquito mortality and blood-feeding status 24 ​h following collection (see the legend in panel **D** for the description of the mosquito status). **B** Breakdown of mosquito numbers in huts with unwashed PermaNet 3.0 nets. **C** Breakdown of mosquito numbers in huts in which unwashed PermaNet 3.0 nets were used. Panels **A**–**C** include pie-charts showing the aggregated mosquito data for each trial arm (as a percentage of total mosquitoes collected in that trial arm). **D** Summary measures over the whole trial for the outcome of a single feeding event by a blood-feeding mosquito: being deterred from entering the hut (green shading, calculated by the difference in the number of mosquitoes caught in the control arm relative to panels **B** and **C**), mosquitoes being alive and unfed, unfed and dead, fed and dead or successfully blood-fed and alive (red, note the percentage is different from pie charts in panels b and c due to the actions of deterrence). **E** and **F** Daily mortality estimates (and 95% confidence intervals, vertical line) for unwashed (**E**) and washed (**F**) PermaNet 3.0 nets, indicating the wide variation observed over the course of the trial. In these panels, the larger circles indicate mortality estimates with narrower confidence intervals (i.e. higher numbers of mosquitoes present). The colour of the points indicates the hut number. The data shown here were reported by Ngufor et al. (2022) from a study carried out in Benin in 2017.

Fig. 1 shows some of the data collected from an EHT carried out in Benin in 2017 (Ngufor et al., 2022). We show individual-level data from three of the study arms: the untreated nets (the control), unwashed PermaNet 3.0 nets, and washed PermaNet 3.0 nets. A much higher proportion of blood-fed mosquitoes were observed in the control arm (52%), along with a very low mosquito mortality (1%). Much higher mosquito mortality was induced in the intervention arms (Fig. 1B and C), particularly in the unwashed PermaNet 3.0 nets. Overall, mosquito counts were much lower in the intervention arms, indicating that in this setting the ITNs had a strong deterrence effect, i.e. the insecticide prevented mosquitoes from entering huts (Fig. 1D) (Sherrard-Smith et al., 2018). High variation in the numbers of mosquitoes entering the huts each night can also be observed (Fig. 1A–C). The high between-observation variability in mortality can be observed in Fig. 1E–F where average mortality varies substantially between nights. In many trials, including this one, this variation cannot be well explained using factors included in the model (the ITN type, the hut, the volunteer), or the amount of variation generated by the binomial sampling process. For this reason, the observation-level random effect has been included in all further analyses. Regarding the deterrence effect, we model this using a negative binomial regression model (see Supplementary file 1). Both East- and West-African experimental huts are surrounded by water-filled moats, to prevent scavenging ants from entering the huts. Ants could otherwise carry off dead or knocked-down mosquitoes, which could bias the estimate of the deterrence effect (WHO, 2013).

A number of software packages can be used to fit the regression model displayed in Equation (1). In the Supplementary file 1, we show a worked example written in R, using the *lme4* package (Bates et al., 2015). A summary of the parameters obtained by fitting the model to a simulated dataset are shown in Equation (1) in Supplementary file 1. The interpretation of these values is not immediately obvious, as they are all expressed on the log-odds scale. They should be converted to a proportion, in order to obtain the estimate of the mortality observed in each trial arm. The standard errors of the estimates of the fixed effects (i.e. $\beta_{m},m=0,\ldots ,6$) can be used to construct 95% confidence intervals for the mortality estimates. In the Supplementary file 1, we also show how to construct an odds ratio (OR), comparing mortality observed in one arm with that observed in another. This quantity will be useful later, when making a non-inferiority assessment.

### 2.2 Assessing superiority

In the context of EHTs, a superiority trial could be used to demonstrate that a novel type of ITN kills significantly more mosquitoes than the current standard of care. Here we consider a trial designed to evaluate whether a next-generation ITN, designed to control pyrethroid-resistant mosquitoes, outperformed a standard pyrethroid-only net. If both unwashed and washed nets are included in the trial, one must decide which assessment(s) to make, e.g. a comparison of the unwashed nets, a comparison of both the unwashed nets and the washed nets, or an assessment of the average induced mortality across the unwashed and washed nets. In the Supplementary file 1 we show a worked example, using the output from the regression model to decide whether the null hypothesis should be rejected (i.e. if the next-generation ITN is deemed superior to the pyrethroid-only ITN). Based upon the trial design outlined above, a statistical test (implemented within the R package *lme4*) is used to determine whether one net is superior to another at a given significance level. As discussed in the *Tutorial* (Supplementary file 1), various statistical tests can be used to do this. As a default, the regression analysis carried out using the *lme4* package uses a Wald test. As this is very quick to calculate, we shall favour this approach when carrying out time-consuming power analyses. In the *Tutorial*, we discuss the limitations of this approach, and how its accuracy can be verified.

In this work, we use PBO-pyrethroid ITNs as an example of a next-generation ITN. When comparing the mortalities of PBO-pyrethroid ITNs to pyrethroid-only ITNs, we will utilise a mathematical relationship between the two, fitted by Sherrard-Smith et al. (2022b). The relationship, calibrated using data from a recent meta-analyses of experimental hut trial data (Nash et al., 2021), states that if the ITN-induced mortality of a pyrethroid-only ITN is l then the mortality induced by a PBO-pyrethroid ITN is given by $1/(1+\exp\left[-\left(\alpha_{1}+\alpha_{2}l\right)\right]$. Here we use the best-fit parameters obtained by Sherrard-Smith ($\alpha_{1}=-1.43$; 95% CrI [−1.54, −1.33], $\alpha_{2}=5.60$; 95% CrI [5.29, 5.93]) (Sherrard-Smith et al., 2022b). We use this result to generate parameters for simulated EHTs. The results are compared to mean mortality estimates recorded in the EHT studies in order to identify potential outliers.

### 2.3 Assessing non-inferiority

**Fig. 2:**
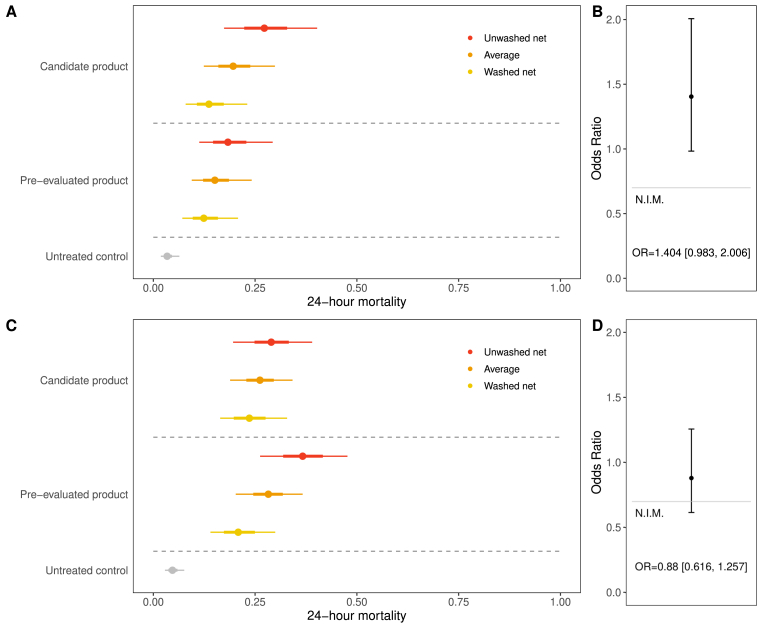
Illustration of the non-inferiority assessment. Here we show estimates of mosquito mortality after 24 ​h in a simulated experimental hut trial. Points (with 68% and 95% confidence interval estimates on the horizontal lines) show unwashed ITN (red), washed ITN (yellow) and an untreated control net (grey). Overall estimate of the ITN mortality is shown in orange and is calculated by combining data from washed and unwashed nets. We show two scenarios, one in which a candidate product is deemed to be non-inferior to an ITN already evaluated in a randomised control trial (top panels: **A**, **B**), and another in which it is not non-inferior (lower panels: **C**, **D**). Both unwashed and washed nets are included in the simulated trials: the averaged mortality across unwashed and washed nets was used to calculate the odds ratio between the two products. The right-hand panels (**B**, **D**) show the odds ratios for the two scenarios, along with their confidence intervals. If the lower confidence interval is greater than the pre-selected non-inferiority margin (NIM), the candidate product is judged to be non-inferior to the pre-evaluated ITN. Here we use an NIM of 0.7, as recommended by the WHO (WHO, 2018). We note that the same underlying parameters were used to generate the two synthetic datasets, illustrating the variation present in the assay.

The methodology used here for evaluating ITNs using a non-inferiority trial design is that proposed by the WHO (WHO, 2018). These trials should include an untreated net, a pyrethroid-only net (referred to here as ‘the standard comparator’), a first-in-class product that has already shown epidemiological impact (‘the active comparator’), and a novel product (‘the candidate net’). Both unwashed and washed ITNs should be included, which means that the trial should have at least 7 arms. The assessment of whether the candidate net is non-inferior to the active comparator is made by calculating the odds ratio (OR) of the mortality induced by these two products. As shown in the Supplementary file 1, we fit a regression model for which data for the unwashed and washed ITNs of the same type are grouped together. The OR is constructed from the parameters estimated from the regression modelling, along with their confidence intervals. For the candidate product to be judged non-inferior to the active comparator, the lower limit of the 95% confidence interval of the OR must be greater than the pre-selected non-inferiority margin (NIM). The NIM is a compromise between the measurement error of the assay and the minimum acceptable epidemiological impact of an inferior product. For mosquito mortality, the WHO have chosen an OR of 0.7 for the NIM. Rather than assessing non-inferiority for the unwashed and washed nets separately, we use the averaged mortality across the unwashed and washed arms to construct the OR. The non-inferiority assessment is illustrated in Fig. 2: the top panels show a scenario in which the candidate product is judged to be non-inferior to the active comparator, and the lower panels outline a scenario where this is not the case (here we would say that the candidate product is ‘not non-inferior’ to the active comparator). In the *Tutorial* (Supplementary file 1), we present a worked example of an assessment of non-inferiority, carried out for simulated EHT data.

### 2.4 Quantifying the uncertainty of experimental hut trials

All EHTs need to be adequately powered to ensure the study can answer the hypothesis under investigation. This is especially the case for non-inferiority trials where the width of confidence intervals strongly influences whether or not a new product is judged to be non-inferior. In order to simulate a trial and estimate study power, it is instructive to look at data from real-world trials to understand the conditions in which they take place. A key factor in the statistical power of any EHT is the number of mosquitoes entering the huts over the course of the trial: the higher this number, the more robust the assay will be. The magnitude of the random effects included in the regression model (Equation (1)) will also play an important role, as they influence the variability present in the assay and hence the width of the confidence intervals of the fixed-effect estimates. These estimates are typically not reported alongside the results of EHT analyses making it difficult to power future studies. Here we examined individual-level data collected by a recent meta-analysis of EHTs (Nash et al., 2021) to better appreciate the numbers of mosquitoes entering huts in these studies, and the magnitude of the random effects observed. The ten most frequently cited authors identified in the EHT meta-analyses were contacted to request the raw data from their trials (number of mosquitoes caught per night and their mortality and blood-feeding status). For each of these trials, we fitted a negative binomial distribution to the number of mosquitoes entering the huts, to obtain estimates for the mean and overdispersion in mosquito counts. A mixed-effects logistic regression was performed on each study separately and the values of $\sigma_{h}$, $\sigma_{k}$ and $\sigma_{b}$ were recorded.

### 2.5 Estimating study power

For a given trial design, statistical power can be estimated using the simulation-based methods (Johnson et al., 2015). By simulating a trial, with parameter values chosen such that the null hypothesis should be rejected, we can determine whether the null hypothesis would be rejected in a single, simulated trial (for a given significance level). By looking at a large ensemble of these simulated trials, an estimate of the power can be generated. Throughout this work, we simulate 1000 trials for a given set of parameter values to provide an estimate of the power.

We use the information on the numbers of mosquitoes entering the huts, and the magnitude of the random effects to simulate EHTs with realistic dynamics. To fully specify the trial to be simulated, we must also define the properties of the nets used. For example, when estimating mosquito mortality, the mortality induced (on average) by the ITN used in each trial arm must be considered, along with the mortality in the control arm. When estimating power for a superiority trial, one must determine a difference in mortality that is considered meaningful. For a non-inferiority trial, one must choose values that lie within the pre-selected NIM, i.e. would lead to the rejection of the null hypothesis. In the non-inferiority analyses carried out here, we simulate ITNs with identical properties although this need not always be the case. In the context of estimating mosquito mortality, this means that the candidate net kills on average the same proportion of mosquitoes as the active comparator.

To simulate trials that include random effects we utilised the R function ‘simm.glmm()’ developed by Johnson et al. (2015). We illustrate how this function can be used for both superiority and non-inferiority trials in the *Tutorial* (Supplementary file 1). For superiority trials we determine whether there is a significant difference (at the 95% significance level) between mortality in the two arms of the trial being considered. For the non-inferiority analyses, we calculate the OR (and its 95% confidence interval) of the mortalities induced by the candidate net and active comparator. We make a non-inferiority assessment with the pre-selected NIM: the statistical power is the proportion of simulated trials for which the candidate net is judged non-inferior, i.e. the correct assessment is made. As this estimate is calculated from a finite proportion, one can also present the uncertainty in the estimate of the statistical power (Johnson et al., 2015), although we do not show this in our results here. We explore the role that the number of mosquitoes collected in each hut and the magnitude of the random effects have on the power, and explore ways to increase the power through increasing the number of observations by either carrying out longer trials or increasing the number of experimental huts used per arm.

We finish this section by describing the default scenario used in the simulations used for the non-inferiority assessments. We simulate trials with one full rotation of the LSD. The control (untreated nets) are assumed to kill 5% of mosquitoes. Whilst unwashed, we assume that both the active comparator and candidate nets kill 50% of the mosquitoes, whilst both the washed nets kill 30%. The number of mosquitoes entering each hut is sampled from a negative binomial distribution with a dispersion parameter equal to 1 (the mean of this distribution will vary across scenarios). The NIM recommended by the WHO is 0.7, expressed as a log-odds ratio (WHO, 2019). We will generate results using this value and will also show how varying the NIM affects a trial’s statistical power.

## 3 Results

Visual inspection of the disaggregated data illustrates the variability in the assay that might not be evident from summary statistics. As an illustration, we show data from an EHT conducted in Benin in 2017 (Ngufor et al., 2022) (Fig. 1). This figure highlights the substantial difference in the numbers of mosquitoes caught per hut per night (across all 7 trial arms, this ranged from 0 to 103, the mean value was 22.8). It also illustrates the substantial between-observation variability in mosquito mortality ($\sigma_{b}^{2}=1.05$), which cannot be explained by other terms in the model. In this study there was little attributable variation between volunteers ($\sigma_{v}^{2}$ < 0.01) or between the different huts ($\sigma_{h}^{2}$ ​= ​0.15).

**Fig. 3:**
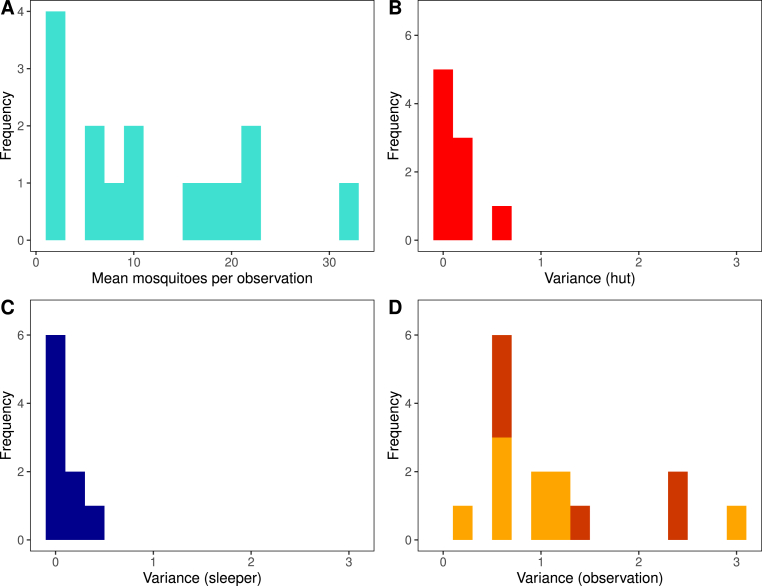
Sources of variability in data from previous experimental hut trials. **A** The mean number of mosquitoes entering each hut each night, across the 15 studies considered here (Supplementary Table S1). **B**-**D** The three sources of variation present in experimental hut trials (huts, sleepers, and observations, respectively). Here we visualise the values of the variances of the random effects in the regression models (Supplementary Table S1). Individual-level information on huts and sleepers was not available for all trials, hence fewer data points are available. This means that some of the observation-level variation recorded for those trials (shown by the darker colour in panel **D**) could be attributed to variation between huts and or sleepers.

Full data were available from 15 different EHTs carried out across sub-Saharan Africa (Supplementary file 1: Table S1) (Ngufor et al., 2014, 2017, 2022; N’Guessan et al., 2016; WHOPES, 2016; Toé et al., 2018; Tungu et al., 2021). There is wide variation in the numbers of mosquitoes entering each hut for studies, with the mean number of mosquitoes per hut varying from 1.6 to 32.5 per night (the median value across the studies was 9.1). In all studies, the count data were overdispersed (median value of dispersion parameter was 1.0). In a trial for which the count data were generated by a process with the median values for the mean and overdispersion, 80% of the mosquitoes would be caught in only 41% of the observations. We found that in general the random effects for hut and sleeper did not explain much of the variation present in the data. However, the observation-level variation in mortality observed per night was often large, with the variance of the associated random effect being greater than 1 in 7 of the 15 studies considered here (Supplementary file 1: Table S1; Fig. 3). Furthermore, the inclusion of this term was supported by a Likelihood Ratio Test (at the 5% significance level) for 14 out of 15 studies (Supplementary file 1: Table S1). This currently unexplained variation means that mosquito mortality can vary substantially from one observation (hut per night) to the next (as illustrated in Fig. 1E–F). The impact of this variation on the overall mortality measured in the trial can be assuaged by the size of the trials. On average, studies ran for a range of 30–73 nights (median 42) and used between 3 and 10 huts (the median was 6). The number of observations for each trial arm ranged from 18 to 100 (median 42).

**Fig. 4:**
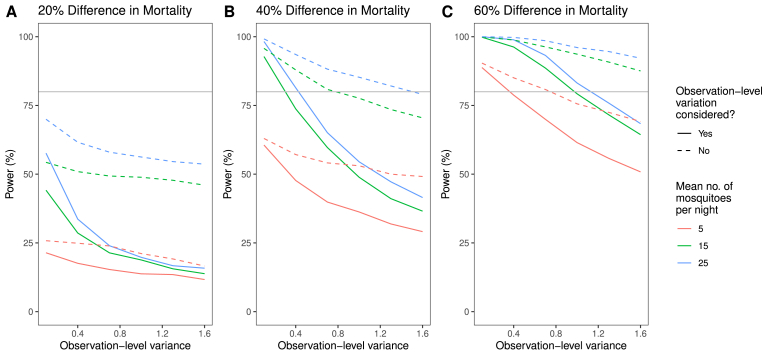
Power estimates for testing for superiority in an experimental hut trial. Here we examine a number of factors that can influence the power of a superiority trial: the difference in mortality between two products, the number of mosquitoes entering the huts and the magnitude of the observation-level variation in the data (characterised by the variance of the corresponding random effect in the GLMM). We considered a scenario in which we tested for superiority of a novel ITN, compared to an ITN with an induced mosquito mortality of 0.25 (i.e. one that would, on average, kill a quarter of all mosquitoes entering a hut in an EHT). We simulated three values for the underlying mortality induced by the novel net: 0.3, 0.35, and 0.4 (panels **A**–**C**, respectively, which state the corresponding percentage difference in induced mortalities between the nets). We varied the mean number of mosquitoes caught per hut per night (fixing the value of the dispersion parameter equal to 2) and the observation-level variation (x-axes), estimating the power using 1000 simulations for each parameter combination (solid lines). The dashed lines show the power estimates that would be obtained if the same synthetic datasets were analysed using a regression analysis without the observation-level random effect (these results can be misleading, as discussed in the *Results* section). All results were generated using one full rotation of a seven-arm trial, containing 343 data points in total (therefore, 49 data points each for the two ITNs evaluated in this scenario). The grey horizontal line in each panel indicates a power of 80%.

We begin our examination of factors that influence the statistical power of a given EHT with a simple example of a superiority trial (Fig. 4). We compare data from two arms of a seven-hut EHT with one full rotation (49 data points per arm, 343 data points in total). We vary the underlying difference between the mortality induced by one net compared to another, as well as the size of the observation-level variation and the number of mosquitoes entering the hut each night. Our results suggest that, for a trial of this duration, there is no guarantee that the trial would be well powered unless the difference between the mortalities induced by the two nets was reasonably large. We also show the results that would be obtained with a regression model that did not contain the observation-level random effect (Fig. 4, dashed lines). These analyses would result in higher power estimates being obtained, primarily because the standard errors for the fixed effects become much smaller, though this approach can give misleading results.

Failure to account for the different sources of uncertainty can substantially over-estimate the precision of the results. We illustrate this in Supplementary file 1: Figure S1, where two estimates for the mortality are obtained from the same simulation, with and without the observation-level random effect. When the observation-level variation is not included, the mortality estimate fluctuates quite appreciably as more data are collected and incorporated into the model. These results have misleadingly narrow confidence intervals. On the other hand, when the observation-level variation is accounted for, the mortality estimate remains more consistent over time, albeit with wider confidence intervals.

**Fig. 5:**
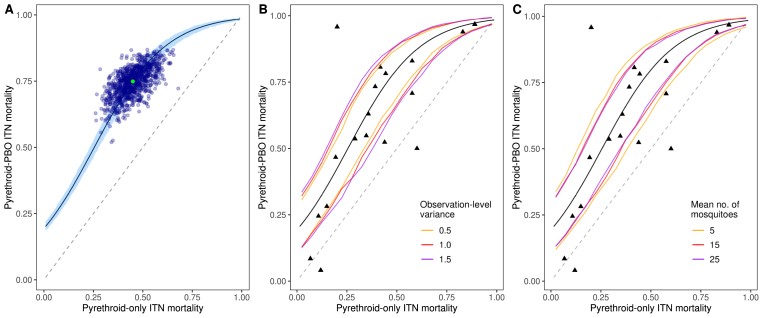
Variability in experimental hut trial results and identification of possible outlier results. Here we use a recently obtained statistical relationship between the mortality induced by pyrethroid-PBO ITNs and pyrethroid-only ITNs (Sherrard-Smith et al., 2022b), fitted to data from experimental hut trials. This relationship is shown by the black curve (shown in all three panels) with the 95% credible intervals for the relationship indicated by the light-blue shaded area in panel **A**. Mosquito mortality is simulated for theoretical hut trials to allows us to illustrate the level of variation present in the assay. **A** We selected one point on the black curve (pyrethroid-PBO mortality equal to 0.75, pyrethroid-only mortality equal to 0.45) indicated by the green dot, and simulated 1000 trials (the mean number of mosquitoes per hut per night was 15, observation-level variance was 1.0). For each trial, point estimates of the observed mortality are shown by the dark blue dots, indicating the variation present in the assay. We next simulated 50,000 more trials, each time randomly selecting the pyrethroid-only mortality and then using the statistical relationship (this time incorporating the uncertainty in the relationship, represented by the light-blue area in panel **A**) to determine the pyrethroid-PBO mortality. We carried out this procedure, varying observation-level variation present in the data (**B**) and the average number of mosquitoes entering each hut (**C**). In panels **B** and **C**, the coloured lines indicate the 95% interval of the measured mortality of the pyrethroid-only and pyrethroid-PBO ITNs, indicating that results become more variable as the observation-level variation increases (**B**) and the mean number of mosquitoes per night per hut decreases (**C**). In panels **B** and **C**, the triangles indicate the observed values used to fit the best-fit curve. Points that fall outside the coloured lines could therefore be considered as outside the range of variability caused by the assay and so could be identified as outliers. All results generated here were from simulations of one full rotation of a seven-arm trial.

We further illustrate the importance of the variation present in the assay on trial results using a mathematical relationship obtained by Sherrard-Smith et al. (2022b), between the mortality induced by pyrethroid-PBO ITNs to that induced by pyrethroid-only ITNs. The latter quantity is used as a measure of the level of pyrethroid resistance in *Anopheles* mosquitoes. The modelled relationship is shown by the black curve in Fig. 5A. Using this relationship between the two mortalities, we simulated EHT data and visualised the outputs obtained. We chose an arbitrary point on the curve (the light green point in Fig. 5A), for which the average mortality due to the pyrethroid nets was 0.45 and the average mortality due to the pyrethroid-PBO nets was 0.75. Simulating 1000 trials (each containing 49 data points for each type of ITN) with the same parameter values, we calculated the central estimates of the two mortalities (the blue dots in Fig. 5A) from the regression model. Due to the variability in the assay, the observed mortality for the pyrethroid-only ITNs varied from 0.293 to 0.599 (95% interval). Similarly, the observed mortality due to pyrethroid-PBO ITNs, which had a true value of 0.75, ranged from 0.61 to 0.85. In these simulations power is sufficiently high for the pyrethroid-PBO ITN to be superior to a pyrethroid-only ITN in a trial of this duration (results not shown). Nevertheless, simulations indicate that the added advantage of the pyrethroid-PBO nets ranged from 32.1 to 122.1% better (the true value was 66.7% better). Similarly, the true difference in the proportion of mosquitoes that died was 0.3 but the 95% range spanned 0.19–0.41. We next varied the pyrethroid mortality between 0.01 and 0.99, calculated the corresponding pyrethroid-PBO mortality using the relationship obtained by Sherrard-Smith et al. (2022b), and generated 50,000 simulated trials. We binned the observed pyrethroid mortalities into 0.01-wide groups and calculated the observed 95% range for the central estimates of the pyrethroid-PBO mortalities for each group (Fig. 5B and C). Increasing both the observation-level variability (Fig. 5B) and decreasing the average number of mosquitoes caught per night (Fig. 5C) increases the width of this range.

The above process allows identification of outlier EHT results while taking into account the uncertainty in the assay. The estimates of mortality, calculated in previous EHTs, used to estimate the average benefit of pyrethroid-PBO ITNs are shown as points in Fig. 5B and C. There is considerable uncertainty between results, with some mortality estimates falling a long way from the best-fit line. Nevertheless, much of this uncertainty can be explained by general variability of the assay. Points that fall outside of this uncertainty shown by the coloured lines could be flagged as possible atypical results and warrant further investigation. Here we show a range of different EHT scenarios, i.e. observational variability (Fig. 5B) or number of mosquitoes per night (Fig. 5C). A more formal analyses should be conducted for each EHT result, taking into account the observed characteristics of the trial for formal outlier identification. Factors that could lead to deviations from the expected behaviour include, but are not limited to: vector species composition, varying mechanisms for pyrethroid resistance, or climatic factors (temperature or humidity). However, investigating these is beyond the scope of this article.

**Fig. 6:**
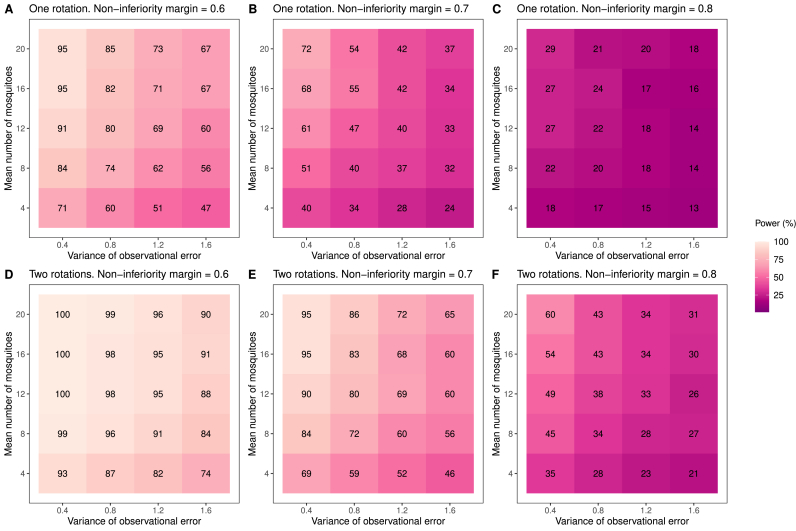
Factors influencing the statistical power of a non-inferiority trial. Here we examined how the number of mosquitoes entering the huts, the magnitude of the observation-level variation in the data (characterised by the variance of the corresponding random effect in the GLMM), and the non-inferiority margin can influence the power of a non-inferiority trial. We assessed whether a candidate net was non-inferior (for mosquito mortality) to the active comparator product, assuming that the two products kill the same proportion of mosquitoes on average (we used a value of 0.3). We varied the mean number of mosquitoes (fixing the value of the dispersion parameter equal to 2) and the observation-level variation, estimating the power using 1000 simulations for each parameter combination. All results were generated using either one full rotation (panels **A**–**C**) or two full rotations (panels **D**–**F**) of a seven-arm trial: one rotation generates 343 data points in total. As illustrated in Fig. 3, the non-inferiority assessment was made from grouping together data from unwashed and washed ITNs of the same type (meaning that each simulated dataset contained 98 data points for each ITN, per rotation).

We now examine the impact that the average number of mosquitoes caught per hut per night and the variability present in the assay can have on the statistical power of non-inferiority trials. We considered a scenario in which the candidate net and active comparator have identical lethality against mosquitoes. Fig. 6 shows how the power of a non-inferiority trial varies with the number of mosquitoes entering the huts and the observation-level variation present in the mosquito mortality. Many of the parameter combinations tested here – which are consistent with those observed in real data – result in trials with low power. In these scenarios, a second rotation of the LSD, or doubling the number of experimental huts used per arm, could be carried out to increase the power. We also show the impact that varying the NIM can have on the power. However, the potential epidemiological impact of decreasing the NIM (or, equivalently, increasing it for measuring blood-feeding inhibition) should be carefully considered.

## 4 Discussion

Experimental hut trials provide a valuable setting in which to evaluate insecticide-treated products, such as ITNs or indoor-residual spraying. As with all biological assays, it is important to be aware of the level of variation present, and the factors that can influence this. In this work, we have summarised how data from EHT can be analysed using GLMMs, to calculate mosquito mortality (or, alternatively, blood-feeding inhibition) due to ITNs. We have built on work due to Johnson et al. (2015), who outlined a framework for using simulation-based methods to generate power estimates in ecological studies. Here we have shown how this approach can be used to estimate statistical power in non-inferiority studies. We have also utilised a recent meta-analysis (Nash et al., 2021) of experimental hut trial, to gain insight into how many mosquitoes enter huts during EHTs, as well as the amount of variation present in the assay (here characterised by the magnitude of the random effects in the GLMMs). We stress that adjusting for the variation present in the assay (variation between huts, sleepers and observations) requires that observation-level data be available for the analysis. If the data are aggregated (e.g. by trial arm) then the full analysis cannot be carried out. Furthermore, visual inspection of the nightly variation in the data allows the identification of possible hypotheses that can be tested further. For example, in the EHT data shown in Fig. 1, in the PermaNet 3.0 arms there is a decline in induced mortality over time that does not appear to be evident in the washed net.

For all trials where data were available, the observation-level variation was larger than the between-hut or between-sleeper variation. It signals that most of this variation cannot be accounted for using the terms currently present in the model. In future work, it would be interesting to see whether other factors could explain this variation (mosquito abundance, environmental temperature, humidity etc.). Understanding this variability present in the assay would increase our understanding of how these interventions work and enable exploration of how public health value might vary between settings. It could also allow more precise estimates of intervention efficacy, increasing the power of studies and potentially allow future trials to be smaller, saving money. In our experience of the literature, power calculations are rarely mentioned in articles that report data from experimental hut trials. We would encourage researchers to consider these when planning such studies, and to state them when reporting the trial results. We hope that the code repository and supplementary *Tutorial* that accompany this article will help this practice become more widespread.

The variability present in the EHT assay can be particularly troublesome when carrying out non-inferiority studies. If, for a given trial, this variability causes the 95% confidence interval for the mosquito mortality (or blood-feeding inhibition) to regularly cross the NIM, it is possible that efficacious non-inferior ITNs could fail to demonstrate non-inferiority to the active comparator product. As shown in Fig. 5, the number of mosquitos entering the huts and the size of the between-observation variation can strongly influence a trial’s power. These quantities cannot be known precisely in advance of the trial, although EHTs previously carried out in the same location can give insight, as can mosquito collections before the trial begins. However, these quantities can be calculated in an interim analysis, without unblinding the trial. Such checks could be carried out, to help determine when the trial can be concluded (i.e. when sufficient power has been reached). Altering the NIM can increase a trial’s statistical power, but this should not be done lightly. If the NIM is too low, it is possible that inferior ITNs can be approved for use (note that, due to the nature of non-inferiority trials, a product can be simultaneously deemed to be inferior and non-inferior, for a given NIM (Schumi & Wittes, 2011). The epidemiological consequences of this should be carefully considered.

The rapid spread of pyrethroid-resistant mosquitoes through sub-Saharan Africa means that next-generation ITNs are urgently needed. The strength of the evolutionary selection pressure for resistance to develop means that any new product, no matter how efficacious, will have a limited time window before their public health impact starts to diminish. The increased cost of next-generation nets means that it is more important than ever that ITNs are regularly rigorously evaluated, to ensure that they are cost-effective. EHTs will continue to play an important role in evaluating insecticidal interventions in the years to come.

## 5 Conclusions

The statistical analyses of EHT data have been standardised and historically appear to be fit for purpose (WHO, 2013; Gleave et al., 2021). Nevertheless, over the years the goals of these trials have shifted from demonstrating entomological impact to quantifying the magnitude of benefit. Gold-standard CRCTs with epidemiological endpoints adhere to very strict standards on design, sample size, good clinical practice, etc., as laid down in the CONSORT checklist (Moher et al., 2010). EHTs have been evaluated against almost all aspects of the CONSORT checklist (WHO, 2013), though it is unclear whether this is routinely conducted and how many studies do rigorous power calculations. If trial results are increasingly to be used in decision-making, then the statistical analyses may need to be refined to ensure results are not overly interpreted. Presenting trial results, alongside power calculations and measurement of nightly trial variability should increase the confidence in the robustness of these important entomological assays and support future evidence-based decision making.

## Funding

This work was supported by the 10.13039/100000865Bill & Melinda Gates Foundation [under Grant Agreement No INV-010445]. JDC, RKN, ESS, and TSC acknowledge funding from 10.13039/501100000265the MRC Centre for Global Infectious Disease Analysis (reference MR/R015600/1), jointly funded by 10.13039/501100000265the UK Medical Research Council (MRC) and 10.13039/501100000265the UK Foreign, Commonwealth & Development Office (FCDO), under the MRC/FCDO Concordat agreement and is also part of the EDCTP2 programme supported by the 10.13039/501100000780European Union. RKN acknowledges funding from 10.13039/501100000265the Medical Research Council (MRC) Doctoral Training Partnership (grant reference MR/N014103/1). ESS is funded by a 10.13039/100014013UKRI Future Leaders Fellowship from the 10.13039/501100000265Medical Research Council (MR/T041986/1). 10.13039/501100000761Imperial College London is grateful for the support of 10.13039/100014013the UKRI Strategic Priorities Fund for supporting this work.

## Ethical approval

Not applicable.

## CRediT author statement

Joseph Challenger: Conceptualisation, Methodology, Formal analysis, Investigation, Software, Visualisation, Writing – original draft, Writing – review & editing. Rebecca Nash: Data curation, writing-review & editing. Corine Ngufor: Resources, Writing – review & editing. Antoine Sanou: Resources, Writing – review & editing. Hyacinthe Toé; : Resources, Writing – review & editing. Sarah Moore: Resources, Writing – review & editing. Patrick Tungu: Resources, Writing – review & editing. Mark Rowland: Resources, Writing – review & editing. Geraldine Foster: Resources, Writing – review & editing. Raphael N’Guessan: Resources, Writing – review & editing. Ellie Sherrard-Smith: Writing – review & editing. Thomas Churcher: Conceptualisation, Methodology, Writing – original draft, Writing – review & editing, Supervision, Project administration, Funding acquisition. All authors read and approved the final manuscript.

## Declaration of competing interests

The authors declare that they have no known competing financial interests or personal relationships that could have appeared to influence the work reported in this paper.

## Data Availability

Data that support the findings of this study are available from the corresponding author upon reasonable request and subject to agreement with the data owners. R code to generate data visualisations from EHTs (as per Fig. 1) is available at: https://github.com/JDChallenger/EHT_Visualise. R code to generate power estimates from EHTs is also available in this repository
