# Supplementary material for: Assessing the variability in experimental hut trials evaluating insecticide-treated nets against malaria vectors
Source: Curr Res Parasitol Vector Borne Dis. 2023 Feb 9;3:100115. doi: 10.1016/j.crpvbd.2023.100115 (PMC9988481; doi:10.1016/j.crpvbd.2023.100115)
Supplement: Multimedia component 1 [file mmc1.pdf]

Assessing the variability in experimental hut trials evaluating insecticide-treated nets against malaria vectors

Joseph D. Challenger[1], Rebecca K. Nash[1], Corine Ngufor[2,3], Antoine Sanou[4], K. Hyacinthe Toé[4], Sarah Moore[5,6,7,8], Patrick K. Tungu[9], Mark Rowland[3], Geraldine M. Foster[10], Raphael N'Guessan[3,11], Ellie Sherrard-Smith[1], Thomas S. Churcher[1]

1. Medical Research Council Centre for Global Infections Disease Analysis, Department of Infectious Disease Epidemiology, Imperial College London, London, United Kingdom

2. Centre de Recherches Entomologiques de Cotonou, Cotonou, Benin

3. London School of Hygiene and Tropical Medicine, London, UK

4. *Centre National de Recherche et de Formation sur le Paludisme, Burkina Faso*

5. Vector Control Product Testing Unit (VCPTU) Ifakara Health Institute, Environmental Health, and Ecological Sciences, Bagamoyo, Tanzania

6. Vector Biology Unit, Department of Epidemiology and Public Health, Swiss Tropical & Public Health Institute, Basel, Switzerland

7. Faculty of Science, University of Basel, Basel, Switzerland

8. The Nelson Mandela African Institution of Science and Technology (NM-AIST), Tengeru, Arusha, Tanzania

9. National Institute for Medical Research, Amani Medical Research Centre, Muheza, Tanzania

10. Vector Biology Department, Liverpool School of Tropical Medicine, Liverpool, United Kingdom

11. Institut Pierre Richet, Bouaké, Côte d'Ivoire

SUPPLEMENTARY MATERIALS

This document contains:

1. **Supplementary Table 1**
2. **Supplementary Figure 1**
3. **Tutorial on analysing experimental hut trial data in R**

References are as per the main text

**Study**	**Trial location and date**	**Arms included**	**Number of data points**	**Number of Huts**	**Mosquito counts per hut per night (mean, *m*, and dispersion, *k*)**	**Time(s) at which mortality recorded**	**Variance of random effect(s)**		
							**Observation** *(p value for Chi-squared test)**	**Hut**	**Sleeper**
N'Guessan et al. (N'Guessan et al. 2016)	Cové, Benin; Oct 2014-Jan 2015	UTN, Chlorfenapyr, Interceptor (0W, 15W, 20W), InterceptorG2 (0W, 15W, 20W)	584	8	*m*=10.3, *k*=1.24	24hrs, 72hrs	0.6 *<2.2e-16*	0.02	-
Ngufor et al. (Ngufor et al. 2014)	Bobo-Dioulasso, Burkina Faso; Aug-Nov 2011	UTN, Permanet2.0 (0W)	108	6	*m*=2.2, *k*=0.34	24hrs	0.69 *0.0113*	0.10	0.13
Toé et al. (Toé et al. 2018)	Bobo-Dioulasso Burkina Faso; Sep-Oct 2014	UTN, DawaPlus2.0 (0W), Olyset (0W), OlysetPlus (0W), Permanet2.0 (0W), Permanet3.0 (0W)	216	6	*m*=32.5, *k*=0.94	24hrs	0.67 *<2.2e-16*	0.18	0.05
Toé et al. (Toé et al. 2018)	Tengrela, Burkina Faso; Sep-Oct 2014	UTN, DawaPlus2.0 (0W), Olyset (0W), OlysetPlus (0W), Permanet2.0 (0W), Permanet3.0 (0W)	216	6	*m*=20.3, *k*=1.42	24hrs	1.21 *<2.2e-16*	0.10	0.04
N'Guessan et al. (WHOPES 2016)	M'Be, Côte d'Ivoire 2015	UTN, alphacyp (1W), MAGNet (0W, 20W), Veeralin (0W, 20W)	216	6*	*m*=16.6, *k*=0.84	24hrs	0.62 *<2.2e-16*	-	-
Unpublished data from Antoine Sanou	Sep-Oct 2019; Banfora, Burkina Faso	UTN, InterceptorG2 (0W, 20W), Permanet2.0 (0W), Permanet3.0 (0W, 20W)	213	6	*m*=17.9, *k*=2.28	24hrs, 72hrs	1.00 *<2.2e-16*	0.14	0.5

Unpublished data from Antoine Sanou	Nov-Dec 2019; Banfora, Burkina Faso	UTN, Interceptor (0W), InterceptorG2 (0W, 20W), Permanet3.0 (0W), RoyalGuard (0W)	123	6	*m*=6.2, *k*=1.03	24hrs, 72hrs	0.26<br>*0.0738*	0.08	0.19
Tungu et al. (Tungu et al. 2021)	Muheza, Tanzania	UTN, Dawa4.0 (0W), Permanet3.0 (0W, 20W), Veeralin (0W, 20W)	216	6	*m*=9.1, *k*=2.79	24hrs	1.32<br>*<2.2e-16*	-	-
Unpublished data from Sarah Moore	Aug-Sep 2018; Ifakara, Tanzania	UTN, DawaPlus (0W, 20W), TsaraNet (0W, 20W),	500	10	*m*=22.0, *k*=0.33	24hrs	0.62<br>*<2.2e-16*	0.02	0.04
Ngufor et al. (Ngufor et al. 2017)	Jun-Sep 2015; Cove, Benin	UTN, Interceptor (0W), InterceptorG2 (0W)	137	3*	*m*=5.4, *k*=2.09	72hrs	2.34<br>*1.55e-15*	-	-
Unpublished data from Mark Rowland	Sep-Dec 2014; Moshi, Tanzania	UTN, chlorfenapyr (0W), Interceptor (0W, 20W), InterceptorG2 (0W, 20W)	301	7	*m*=1.6, *k*=0.84	72hrs	2.96<br>*9.98e-08*	0.52	0.09
Unpublished data from Mark Rowland	Dec 2015-Feb 2016; Muheza Tanzania	UTN, chlorfenapyr (0W), Interceptor (0W, 20W), InterceptorG2 (0W, 20W)	324	6	*m*=1.8, *k*=0.84	72hrs	1.28<br>*0.000101*	0.03	0.06
Unpublished data from Mark Rowland	Muheza, Tanzania	UTN, chlorfenapyr (0W), Interceptor (0W, 20W), InterceptorG2 (0W, 20W)	216	6*	*m*=2.4, *k*=0.93	72hrs	2.43<br>*4.51e-09*	-	-
Ngufor et al. (2022) (Ngufor et al. 2022)	Cove, Benin; Feb-April 2017	UTN, Olyset (0W, 20W), OlysetPlus (0W, 20W), Permanet 3.0 (0W, 20W)	294	7	*m*=22.8, *k*=1.6	24hrs	1.05<br>*<2.2e-16*	0.15	<0.01
Unpublished data from Mark Rowland	Benin 2014.	UTN, chlorfenapyr (0W), Interceptor (0W, 20W), InterceptorG2 (0W, 20W)	258	6*	*m*=7.1, *k*=1.66	72hrs	0.56<br>*8.80e-11*	-	-

Supplementary Table 1: Summary of the experimental hut trials analysed in this study. For each study, we list the ITNs evaluated, time period of data collection, the number of data points recorded, and the number of huts used. For the mosquito count data, we first checked whether the data from each study was better described by a Poisson or negative-binomial distribution (all count data were better described by the latter). The proportion of mosquitoes killed in each arm of a study was assessed using a GLMM (Methods). If a dataset contained individual-level data on the corresponding hut and sleeper for each data point, hut and sleeper were included as random effects. If not, observation was the only random effect included and, in those studies, may include variation due to huts and sleepers. For datasets that assessed mosquito mortality at two time points, the later time point was used for the regression modelling. *Individual-level information on huts and sleepers not available for these studies. The number of washes is denoted by 0W or 20W for zero or twenty washes, respectively. Arithmetic means are provided, with lower values of *k* indicating more overdispersed count data.

* P-value obtained from a Likelihood Ratio Test, test statistic follows a chi-squared distribution with 1 degree of freedom.

Supplementary Figure 1: Simulated experimental hut trial showing estimates of mortality due to an ITN with and without accounting for the observation-level random effect. In this scenario, we simulated an experimental hut trial, with observation-level variation ($\sigma_b^2 = 1$) present in the data-generating process of strength comparable to past studies (Figure 3, Supplementary Table 1). We calculated two estimates of the mosquito mortality (with 95% confidence intervals), using a fixed-effects model (red) and a mixed-level model, containing an observation-level random effect (blue). For unlimited data, the two estimates should align: for small dataset, the differences are quite apparent. The estimate from the fixed-effects model has much narrower confidence intervals which, in this example, do not always contain the true value of mosquito mortality used in the simulation (0.5, indicated by the grey line). In contrast, the estimate from the multi-level regression model has much wider confidence intervals and is more consistent with the true value of the mortality parameter. In this scenario, data was generated for a one-hut trial, so no between-hut or -sleeper variation was generated. Note that the number of data points considered (nights of mosquito collection, x axis) is shown on a log scale. The number of mosquitoes entering the hut each night followed a negative binomial distribution with a mean of 10 and a dispersion parameter equal to 2.

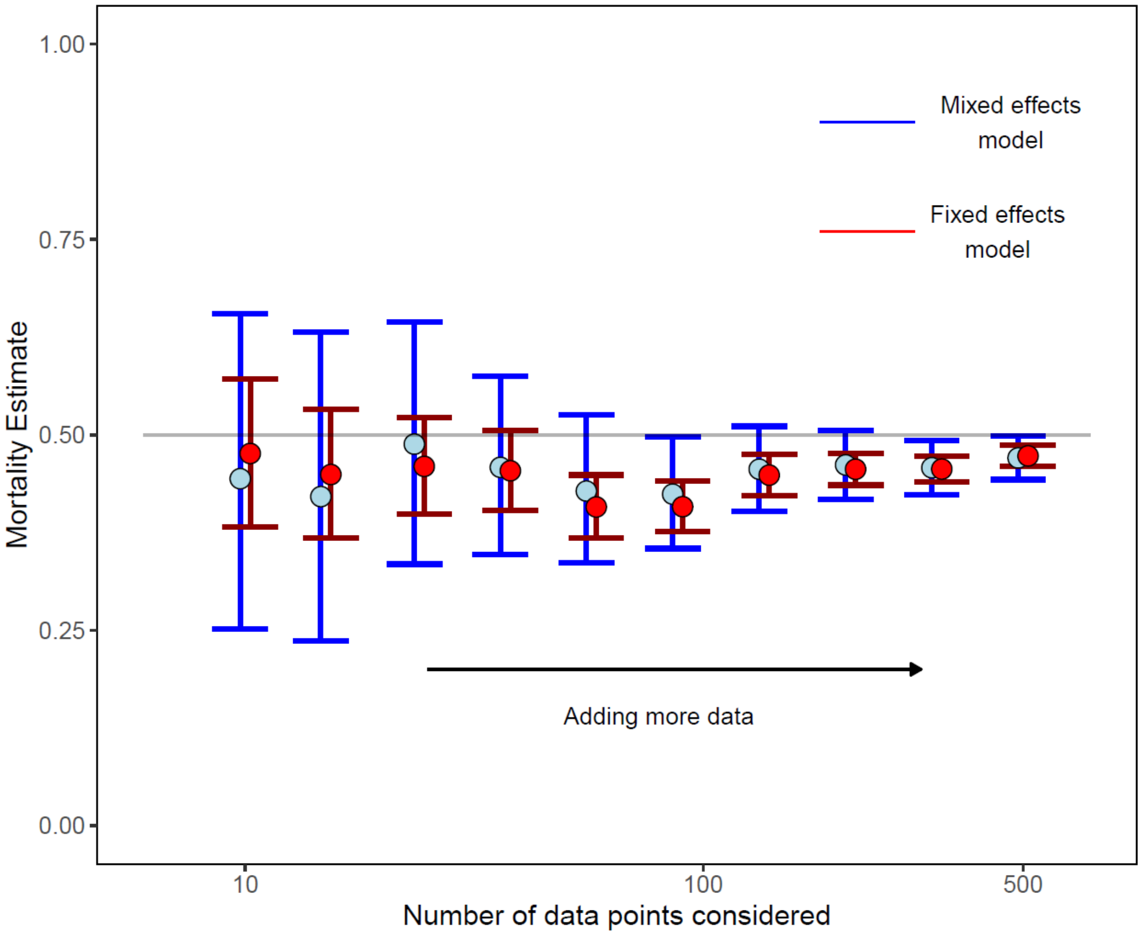

Tutorial: Analysing data from experimental hut trials

A brief introduction to R

The code included in this tutorial is written in R. We do not include a comprehensive introduction to R, as many others are available elsewhere. However, we will include a few brief comments on the syntax of R. Variables (be they numbers, or character strings) can be stored in the internal memory using either `=` or `<-`. For example, writing `x<-5` assigns a value of 5 to `x`. The symbol `#` is used to indicate a comment. That is, anything that follows a `#` will not be read as R code.

R contains a number of core functions, which carry out commonly used operations. Additional functions can be found within *packages*. A package can be loaded using the `library()` function. For example, we can load the ggplot2 package, which is a versatile library for making graphs, by running the following command:

```
> library(ggplot2)
```

If you have not used this package before, you may need to download it. You can do this by running the command `install.packages('ggplot2')`. Alternatively, if you're using Rstudio, you can click on the 'Tools' menu, then click on 'Install packages...', and search for the desired package.

1 Loading & summarising a dataset

In this document, we will demonstrate how to carry out the statistical analyses discussed in the main manuscript. To demonstrate these concepts, we will use a simulated dataset[1]. This dataset has been uploaded with these materials, along with an R script containing the work outlined in this tutorial. To run the R script you should download R & RStudio. If you wish to run the script, you should download the ZIP file, and extract the folder. Then, double-click on the project file `EHT_Visualise.Rproj` to open it in RStudio.

Once the R project file is open, you can then open the R script `script_for_tutorial.R`. The dataset has been stored as an .rds file, and can be loaded using the following command:

```
> df <- readRDS('data_for_plot.rds')
```

Let's take a look at the data:

```
> dim(df)
[1] 343  10
```

[1] The code and data used here can be found at: https://github.com/JDChallenger/EHT_Visualise.

```
> head(df)
  day hut sleeper treatment total unf_live unf_dead bf_live bf_dead tot_dead
1   1   1       2         C     9        7        0       2       0        0
2   1   2       3       N1u     3        3        0       0       0        0
3   1   3       4       N1w     3        3        0       0       0        0
4   1   4       5       N2u     5        3        1       0       1        2
5   1   5       6       N2w    12       11        1       0       0        1
6   1   6       7       N3u    32       16        8       6       2       10
```

For this experimental hut trial (EHT), 343 data points were collected. For each of these, the total number of mosquitoes entering each hut each night was recorded, as well as their mortality & blood-feeding status (`unf_live` = unfed & alive; `unf_dead` = unfed & dead; `bf_live` = blood fed & live; `bf_dead` = blood fed & dead). As the following commands tell us, the trial contained 7 different nets, 7 volunteers, and 7 huts:

```
> table(df$treatment)

  C N1u N1w N2u N2w N3u N3w
 49  49  49  49  49  49  49
> table(df$hut)

 1  2  3  4  5  6  7
49 49 49 49 49 49 49
> table(df$sleeper)

 1  2  3  4  5  6  7
49 49 49 49 49 49 49
```

This EHT contains an untreated net (labelled 'C' for control) and three insecticide-treated nets (ITNs), labelled `N1`, `N2` and `N3`. The suffix 'u' indicates an unwashed (*i.e.* new) ITN; the suffix 'w' indicates a washed (*i.e.* aged) ITN.

2 Visualising the EHT data

Figure 1 shows some output from 3-arms of the simulated trial, namely the untreated control, and both unwashed and washed `N1` nets. The R code that accompanies this document shows how these panels may be generated.

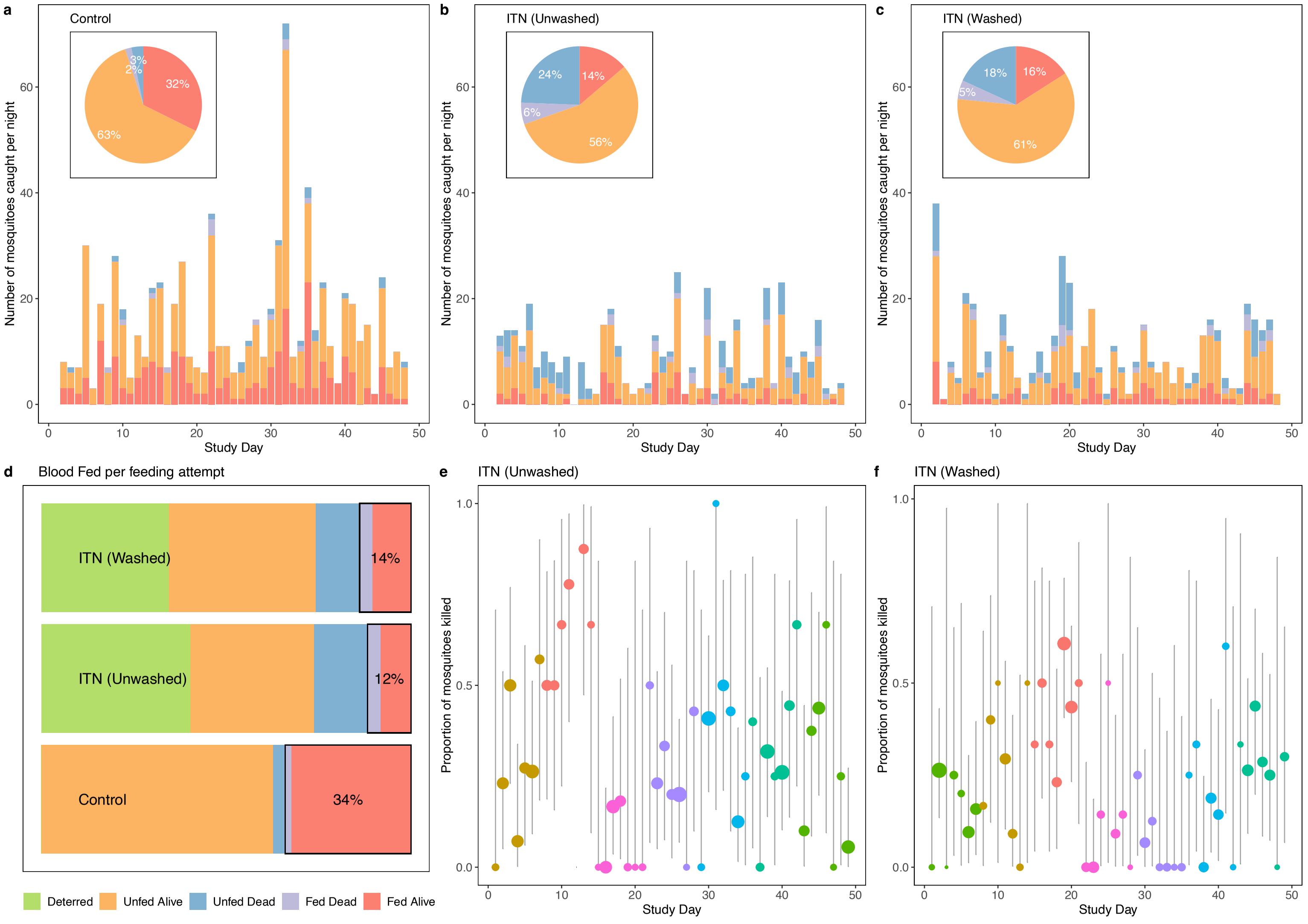

Figure 1: Visualisation of the simulated EHT data. Here we show output from 3-arms of the simulated trial, namely the untreated control, and both unwashed and washed N1 nets

3 Fitting a regression model to the data

We'll now show how to fit the regression model shown in Equation 1 in the main text. As this model contains an observation-level random effect, we first need to provide a unique indicator variable for each row in the data frame *e.g.*

```
df$observation <- factor(formatC(1:nrow(df), flag="0", width=3))
```

For mortality, we

```
>fit <-
  glmer(
    cbind(tot_dead, total - tot_dead) ~
      treatment + (1 | hut) + (1 | sleeper) + (1 | observation),
    family = binomial, data = df)
```

This is equivalent to the equation we wrote down in the main text, which we also include here, for convenience:

$$\log\left(\frac{p_{ijk}}{1-p_{ijk}}\right) = \beta_0 + \sum_m \beta_m + b_i + h_j + v_k. \tag{1}$$

Here we characterise each data point using the subscripts (i, j, k). Subscript i uniquely identifies each data point, j indicates the hut used, k indicates the volunteer sleeping under the net. Then we run the command `summary(fit)` to examine the fitted model. This output is quite lengthy: here we will focus on the fixed and random effects. The former will look like this:

```
Fixed effects:
             Estimate Std. Error z value Pr(>|z|)
(Intercept)   -3.1093     0.2533 -12.276  < 2e-16 ***
treatmentN1u   2.2484     0.2331   9.647  < 2e-16 ***
treatmentN1w   1.7157     0.2354   7.290 3.11e-13 ***
treatmentN2u   2.1026     0.2327   9.034  < 2e-16 ***
treatmentN2w   1.4804     0.2358   6.280 3.40e-10 ***
treatmentN3u   2.3913     0.2314  10.332  < 2e-16 ***
treatmentN3w   1.5985     0.2356   6.784 1.17e-11 ***
```

An estimate for each of the fixed effects is provided, along with its standard error, which indicates the level of precision with which each parameter has been estimated. The z & p values relate to testing whether the parameter estimates are significantly different from 0. It is not immediately obvious how these parameter values relate to our problem: working out the proportion of mosquitoes killed (or blood fed) in each trial arm. This is because a binomial regression like this has been carried out by transforming the proportions onto the log-odds scale. This transformation maps the interval [0,1] (used for proportions, prevalence, or probabilities) to the interval $[-\infty, \infty]$. This removes the difficulty caused by the fact that the probability scale (which runs from 0 to 1) has a floor and ceiling *i.e.* values below 0 and above 1 are forbidden.

The estimated values of the random effects will be presented like this:

```
Random effects:
 Groups      Name        Variance Std.Dev.
 observation (Intercept) 0.24663  0.4966
 sleeper     (Intercept) 0.04405  0.2099
 hut         (Intercept) 0.14988  0.3871
Number of obs: 343, groups:  observation, 343; sleeper, 7; hut, 7
```

By definition, all the random effects are normally distributed with zero mean. Therefore, they are fully characterised by the values of the variance (or, equivalently, the standard deviation). **Aside:** The observation-level random-effect is included in order to account for any overdispersion in mosquito mortality. To check whether this term needs to be included, we can look at the model that drops this term, and compare the two models using a Likelihood Ratio Test (LRT). The simpler model `fit0` is:

```
>fit0 <-
  glmer(
    cbind(tot_dead, total - tot_dead) ~
      treatment + (1 | hut) + (1 | sleeper),
    family = binomial, data = df)
```

The LRT is carried out using the anova function:

```
> anova(fit,fit0)
Data: df
Models:
fit0: cbind(tot_dead, total - tot_dead) ~ treatment + (1 | hut) + (1 | sleeper)
fit: cbind(tot_dead, total - tot_dead) ~ treatment + (1 | hut) + (1 | sleeper) + (1 | observa
     npar    AIC    BIC  logLik deviance Chisq Df Pr(>Chisq)
fit0    9 1141.4 1175.9 -561.70   1123.4
fit    10 1117.7 1156.1 -548.87   1097.7 25.67  1  4.051e-07 ***
```

We consider a p value 'Pr(>Chisq)' <0.05 to indicate a signficant improvement in model fit. Therefore, we use the complicated model in our analyses. The same is true in almost all of the real EHT datasets (Supplementary Table 1). **End of Aside**

In order to understand the output of the regression model, we will use the inverse of this log-odds transformation. If $X(p)$ denotes the log-odds-transformed proportion, we write

$$X(p) = \log\left(\frac{p}{1-p}\right).$$

Note that, on the log-odds scale, values can be positive or negative. A value of 0 corresponds to $p = 0.5$. We can write the inverse function like this:

$$p = \text{InvLogit}(X) = \frac{\exp(X)}{\exp(X)+1}$$

Figure 2 shows this function, which approaches 1 as $X \to \infty$, and approaches 0 as $X \to -\infty$. In R, we will define a function to carry out this transformation, as we will be using it frequently. We write

```
InvLogit <- function(X){
  exp(X)/(1+exp(X))
}
```

Let's use this function to find the mortality in the control arm (where untreated nets were used). This is indicated by the intercept in the regression model.

```
InvLogit(coef(summary(fit))["(Intercept)", "Estimate"])
0.04272482
```

Furthermore, we can use the standard error to create a 95% confidence interval. We do this by adding or subtracting 1.96× the standard error, before converting to a probability:

```
InvLogit(coef(summary(fit))["(Intercept)", "Estimate"]+
c(-1,1)*1.96*coef(summary(fit))["(Intercept)", "Std. Error"])
[1] 0.02644880 0.06831393
```

Let's now estimate the mortality in one of the arms containing an ITN. To find the estimated mortality due to unwashed 'N1' nets we perform this calcualation:

```
InvLogit(coef(summary(fit))["(Intercept)", "Estimate"] +
coef(summary(fit))["treatmentN1u", "Estimate"])
[1] 0.2971546
```

This follows from Eq, 1: we have to add the value of the fixed effect parameter to the intercept (β_0) before applying the transformation to the probability scale. We can also generate confidence intervals for this estimated mortality. Strictly speaking we should use the standard errors of both parameter estimates here, and respect the fact that they may be correlated with each other. The variance-covariance matrix for the fixed-effects parameters can be accessed with the `vcov()` function. To calculate the mortality of the unwashed N1 ITN, we had to add together two regression parameters. We can use the standard errors to calculate the standard error in their sum (using results for normally-distributed random variables).

```
rho1_2 <- vcov(fit)[1,2]/(sqrt(vcov(fit)[1,1])*sqrt(vcov(fit)[2,2]))
sigma1_2 <-  sqrt(vcov(fit)[1,1] + vcov(fit)[2,2] +
                  2 * rho1_2 *(sqrt(vcov(fit)[1,1]) *(sqrt(vcov(fit)[2,2]))))
```

The R code in the repository that accompanies this article contains some useful functions to summarise the mortality estimates and their confidence intervals.

For some analyses, we will want to group washed & unwashed nets of the same type together. We do this by introducing a new variable 'net'.

```
> df$net <- NA
> df[df$treatment==‘C’,]$net <- ‘C’
> df[df$treatment==‘N1u’,]$net <- ‘N1’
> df[df$treatment==‘N1w’,]$net <- ‘N1’
> df[df$treatment==‘N2u’,]$net <- ‘N2’
> df[df$treatment==‘N2w’,]$net <- ‘N2’
> df[df$treatment==‘N3u’,]$net <- ‘N3’
```

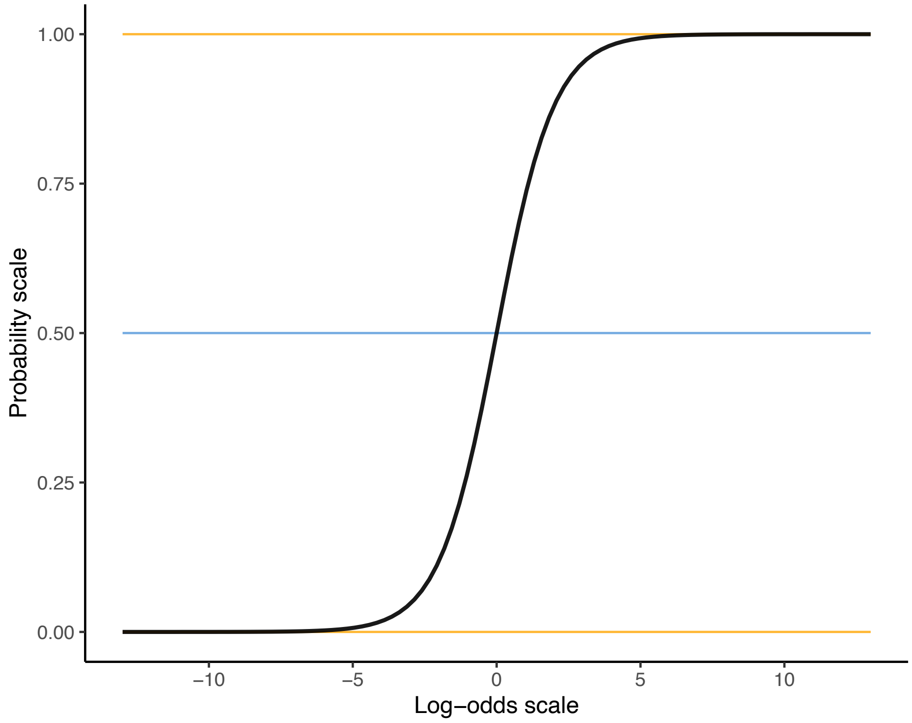

Figure 2: Relationship between the log-odds and probabilty scales. The function is symmetric about 0, which corresponds to a probability of 0.5. The probability approaches 1 as the log-odds value becomes very large (technically, we say ‘as it tends to infinity’). Similarly, the probability goes to 0 as the log-odds value tends to minus infinity.

```
> df[df$treatment==‘N3w’,]$net <- ‘N3’
> table(df$net, useNA = ’a’)

   C   N1   N2   N3 <NA>
  49   98   98   98    0
```

We can then fit this model like this:

```
> fit_n <-
+   glmer(
+     cbind(tot_dead, total - tot_dead) ~
+       net + (1 | hut) + (1 | sleeper) + (1 | observation),
+     family = binomial, data = df)
```

You can use the `summary()` function to analyse the output.

4 Deterrence

In EHTs, it is often observed that fewer mosquitoes are collected in huts containing ITNs, compared to those containing untreated nets. This phenomenon is known as ‘deterrence’. We

can examine whether or not this is the case for our dataset by calculating the mean number of mosquitoes observed across all the different trial arms:

```
tapply(df$total, df$treatment, mean)
        C       N1u       N1w       N2u       N2w       N3u       N3w
15.408163  9.204082 10.102041 10.163265 11.428571  9.142857 11.122449
```

In this EHT, it appears that fewer mosquitoes entered huts containing ITNs, compared with those containing the untreated nets. We will use a different type of regression model to examine this. This is because we will be dealing with mosquito count data, rather than looking at proportions (*e.g.* proportion of mosquitoes killed or blood fed). Count data can be analysed by using a Poisson distribution, or a two-parameter distribution, such as the negative binomial. The latter is used when the count data are *overdispersed*, *i.e.* the standard deviation of the data is greater than its mean value. It is possible to perform a statistical test (see R script `script_for_tutorial.R`, Section 4), to see which distribution to use. However, in our experience with EHT trials, the data are almost always overdipsersed. Therefore, we will use the negative binomial distribution in this document. We perform a negative binomial regression using the function `glmer.nb()`, also from the `lme4` package.

```
fit_nb <- glmer.nb(total ~ treatment + (1|hut), data = df)
summary(fit_nb)
```

(Note that here we've dropped the random-effect for 'sleeper', as it produced a singular model fit, with a estimated variance very close to 0. Let's look at the (truncated) model output:

```
Random effects:
 Groups Name        Variance Std.Dev.
 hut    (Intercept) 0.01059  0.1029
Number of obs: 343, groups:  hut, 7

Fixed effects:
             Estimate Std. Error z value Pr(>|z|)
(Intercept)    2.7263     0.1057  25.784  < 2e-16 ***
treatmentN1u  -0.5126     0.1420  -3.609 0.000307 ***
treatmentN1w  -0.4113     0.1415  -2.908 0.003642 **
treatmentN2u  -0.4239     0.1416  -2.993 0.002758 **
treatmentN2w  -0.2937     0.1406  -2.089 0.036717 *
treatmentN3u  -0.5212     0.1421  -3.668 0.000244 ***
treatmentN3w  -0.3291     0.1408  -2.337 0.019446 *
```

Note that all the fixed-effect parameters are negative, since fewer mosquitoes enter huts containing an ITN, relative to the control. To understand the numerical values presented here, we must appreciate that the counts have been log-transformed. If we take the exponential of the intercept we find:

```
exp(coef(summary(fit_nb))["(Intercept)", "Estimate"])
[1] 15.27638
```

This value is extremely close to the mean mosquito count found in the control arm (see above). The regression model will also return an estimate of the dispersion parameter:

```
getME(fit_nb, "glmer.nb.theta")
[1] 2.464817
```

Let's now calculate the mean number of mosquitoes per night in huts contained unwashed N1 nets:

```
exp(coef(summary(fit_nb))["(Intercept)", "Estimate"]+
coef(summary(fit_nb))["treatmentN1u", "Estimate"])
[1] 9.149449
```

We can use these values to estimate the deterrence effect in terms of a percentage:

```
mean_C <- exp(coef(summary(fit_nb))["(Intercept)", "Estimate"])
mean_E1 <- exp(coef(summary(fit_nb))["(Intercept)", "Estimate"]+
100*(1-mean_E1/mean_C)
[1] 40.10722
```

5 Superiority trials

Frequently in EHTs, we are interesting in seeing if one type of bednet is superior to another, in terms of *e.g.* mosquito mortality or blood-feeding inhibition. This could be a comparison between an ITN and the untreated control, or a comparison between two ITNs. When the logistic regression model is fitted in lme4, a p value for a Wald Z-test is returned, along with the estimates of the value of each fixed effect. This p value stems from a null hypothesis that the value of the fixed effect is 0 (or, alternatively, the mortality odds ratio for a particular ITN relative to the untreated control is equal to 1). Let's recap the output for the fixed-effect parameters shown in the previous section:

```
Fixed effects:
             Estimate Std. Error z value Pr(>|z|)
(Intercept)   -3.1093     0.2533 -12.276  < 2e-16 ***
treatmentN1u   2.2484     0.2331   9.647  < 2e-16 ***
treatmentN1w   1.7157     0.2354   7.290 3.11e-13 ***
treatmentN2u   2.1026     0.2327   9.034  < 2e-16 ***
treatmentN2w   1.4804     0.2358   6.280 3.40e-10 ***
treatmentN3u   2.3913     0.2314  10.332  < 2e-16 ***
treatmentN3w   1.5985     0.2356   6.784 1.17e-11 ***
```

Here we can see whether differences in mortality between the untreated control and any of the ITNs are judged to be significant. However, it is not immediately obvious how to test for superiority of one ITN compared to another. The simplest way to proceed is to change which net is chosen as the intercept in the regression model. By default, `lme4` will choose the first value, ordering the options alphabetically. One way to change the trial arm used for the intercept is to rename one of the treatment arms, so that it becomes the first category (*i.e.* in alphabetical order) e.g. `df[df$treatment=='N1u',]$treatment <- 'A'`. However, it would be better to avoid this, as re-labelling the trial arms can introduce confusion. One way forward is to make treatment a factor variable in R, and modify the order of the levels in the factor. Suppose that we wish to test whether unwashed N2 nets (`N3u`) are superior to unwashed N1 nets (`N2u`). We begin by making the latter the 1st level in the factor,

```
df$treatment <- as.factor(df$treatment)
#First check the current levels of the factor
levels(df$treatment)
> levels(df$treatment)
[1] "C"   "N1u" "N1w" "N2u" "N2w" "N3u" "N3w"
#Make unwashed N1 nets the intercept category
df$treatment <- relevel(df$treatment,"N2u")
#check the levels again
levels(df$treatment)
> levels(df$treatment)
[1] "N2u" "C"  "N1u" "N1w"  "N2w" "N3u" "N3w"
```

Once this has been carried out, we can run the regression analysis again, and find the desired p value. The section of the model output relating to the fixed-effects now looks like this:

```
Fixed effects:
             Estimate Std. Error z value Pr(>|z|)
(Intercept)   -1.0068     0.2142  -4.701 2.59e-06 ***
treatmentC    -2.1026     0.2327  -9.034  < 2e-16 ***
treatmentN1u   0.1459     0.1901   0.768  0.44277
treatmentN1w  -0.3868     0.1953  -1.981  0.04757 *
treatmentN2w  -0.6222     0.1950  -3.190  0.00142 **
treatmentN3u   0.2887     0.1885   1.531  0.12574
treatmentN3w  -0.5040     0.1938  -2.601  0.00930 **
```

Notice that the P value for the fixed effect `treatmentN3u` is not signficant (>0.05). By default, the `glmer()` model will output p values for a Wald z test. We will use this for our power analysis, as it is the quickest test to perform. The Wald test assumes that the log-likelihood is quadratic. Alternatively, we could use a Likelihood Ratio Test (LRT). To illustrate this simply, let's look at a subset of the data, pertaining only to the two trial arms in question (`N2u`,`N3u`):

```
df2 <- df[df$treatment=='N2u'|df$treatment=='N3u',]
```

Let's look at the 'full model', which assumes a fixed effect is needed *i.e.* mosquito mortality is different across the two trial arms. On this smaller dataset, we've just included one of the random-effects, for simplicity:

```
    fit2 <-
  glmer(
    cbind(tot_dead, total - tot_dead) ~
      treatment + (1 | observation),
    family = binomial, data = df2)
```

The simpler model assumes that the fixed effect is not needed

```
fit3 <-
  glmer(
    cbind(tot_dead, total - tot_dead) ~
```

```
    (1 | observation),
  family = binomial, data = df2)
```

We run the anova function (`anova(fit2,fit3)`), which returns the following output:

```
> anova(fit2,fit3)
Data: df2
Models:
fit3: cbind(tot_dead, total - tot_dead) ~ (1 | observation)
fit2: cbind(tot_dead, total - tot_dead) ~ treatment + (1 | observation)
     npar    AIC    BIC  logLik deviance  Chisq Df Pr(>Chisq)
fit3    2 367.42 372.59 -181.71   363.42
fit2    3 367.31 375.06 -180.66   361.31 2.1151  1     0.1459
```

This returns a p value for a chi-squared test (if the null hypothesis is rejected, the more complicated model–fit2– is justified). In this instance, the Wald test & the LRT are in agreement that there is not a significant difference between the mosquito mortality induced by unwashed N2 nets and unwashed N3 nets. If we did find discordance between the two tests, we could investigate further using bootstrapping to more precisely estimate the 95% confidence interval for the fixed effect:

```
> confint(fit2, method = "boot", nsim = 1000, parm = "beta_")
Computing bootstrap confidence intervals ...
                  2.5 %     97.5 %
(Intercept)  -1.3362054 -0.6975908
treatmentN3u -0.1114323  0.7553065
```

Again this calculation indicates that the confidence interval for the fixed-effect cross zero (*i.e.* not significant). Note, however, that this procedure takes much longer to perform.

To examine whether the confidence intervals for a parameter estimate are symmetrical about the central estimate, we can plot the LRT statistic with respect to the fitted parameter(s). The test statistic here is the change in the model deviance, which is -2 times the log-likelihood. If the confidence intervals for a parameter estimate are symmetric, the LRT statistic should be quadratic with respect to the parameter in question. Here we can use the `profile()` function, which returns the square root of the LRT statistic, since it is more straightforward to check whether the plot follows a straight line (compared to a quadratic curve). To this end, `profile()` also returns the negative square root to the left of the parameter estimate. We can view the fixed effects in model `fit2` using the following commands (see Figure 3A)

```
trp <- profile(fit2, which = 'beta_')
lattice::xyplot(trp)
#lattice::xyplot(trp, absVal = TRUE) #This would omit the negative square
#root to the left of the parameter estimate
```

The plotted quantity for each parameter (indicated with ζ in Figure 3A) can be compared with the standard normal distribution. This means that the profile plots can be converted to density plots. We'll demonstrate this using the parameter for `N3u`:

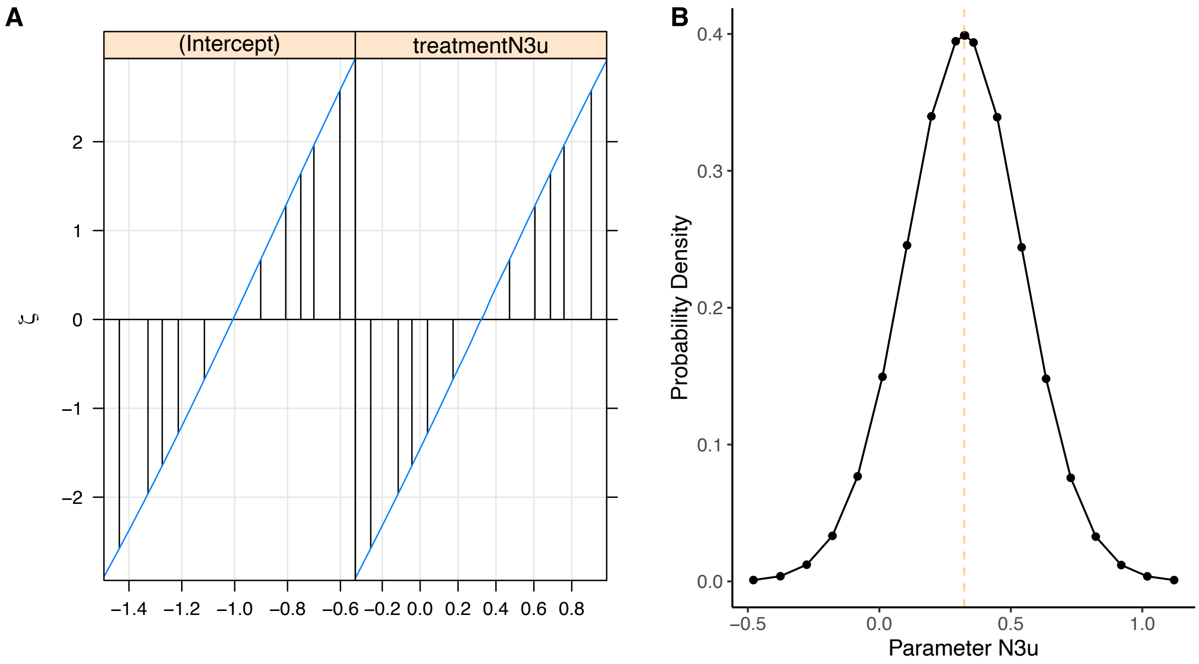

Figure 3: Profile plots & probability density of a parameter estimate. Panel A: The signed square root of the LRT statistic, for a GLMM of data subset containing only two trial arms (N2u, N3u). These curves closely resemble straight lines, indicating that the confidence intervals for these parameters should be symmetric. Panel B: The equivalent density plot for the value of parameter N3u. Parameter ζ, plotted in panel A, can be compared to a standard normal distribution. This means that we can use the plot for ζ to find the distribution for the parameter estimates. Here we illustrate this using the fixed-effect parameter N3u.

```
#First we extract the relevant data:
dfr <- data.frame('p' = trp[trp$.par=='treatmentN3u',]$treatmentN3u,
                  'sl' = trp[trp$.par=='treatmentN3u',]$.zeta)
# convert \zeta to a density function
dfr$dens <- dnorm(dfr$sl,mean=0,sd=1)
#Here's the plot, with the central estimate (dashed orange line) taken
#from the regression model
ggplot(dfr, aes(x=p,y=dens)) + geom_line() + geom_point() + theme_classic() +
  xlab('Parameter N3u') + ylab('Probability Density') +
  geom_vline(xintercept = coef(summary(fit2))["treatmentN3u", "Estimate"],
             color = 'orange', linetype = 'dashed', alpha = .5)
```

The density function is plotted in Figure 3B. **To summarise this section**: the simple Wald test is quick to perform, and should perform well enough for power estimates, in which a large number of simulated datasets need to be assessed. When presenting real data from an EHT, however, the assumptions underlying the Wald test should be checked, either using bootstrapping estimates, or via a LRT, as shown here.

6 Non-inferiority trials

In this section, we show how to use the output of the regression model to make an assessment of whether one ITN is non-inferior to another (*e.g.* in terms of mosquito mortality or blood-feeding inhibition). In order to do this, a non-inferiority margin (NIM) must be selected. This should be chosen before the trial is started. According to World Health Organisation guidelines, the non-inferiority assessment should be made based on the odds ratio of the two products, and a NIM of 0.7 should be used for mosquito mortality (1.43 for blood-feeding inhibition). As with superiority trials, it is more straightforward if the comparator net is the reference category in the regression model. Let's test whether the ITN N3 is non-inferior to N2. We will use the `net` variable we defined earlier for this. In other words, we are comparing the mortality averaged across washed and unwashed nets of the same type. (Note: this should only be done if there are an equal number of data points in the washed and unwashed arms. Otherwise, the two arms will be unfairly weighted.) Let's ensure that the `net` variable is a factor variable and make N2 the default category (*i.e.* the intercept).

```
df$net <- as.factor(df$net)
#check the levels
levels(df$net)
#relevel
df$net <- relevel(df$net,"N2")
#check the levels again
levels(df$net)
```

Now, if we run the regression model, the output for the fixed-effects should look like this:

```
Fixed effects:
            Estimate Std. Error z value Pr(>|z|)
(Intercept)  -1.3314     0.1977  -6.734 1.65e-11 ***
netC         -1.8128     0.2218  -8.174 2.98e-16 ***
netN1         0.1905     0.1457   1.307    0.191
netN3         0.2065     0.1444   1.430    0.153
```

The odds ratio of the mortality induced by N3 compared to N2 is:

```
exp(coef(summary(fit_n))['netN3','Estimate'])
[1] 1.229333
```

Then, we use the standard error to find the 95% confidence intervals

```
> exp(coef(summary(fit_n))['netN3','Estimate'] -
1.96*coef(summary(fit_n))['netN3','Std. Error'])
[1] 0.9263213
> exp(coef(summary(fit_n))['netN3','Estimate'] +
1.96*coef(summary(fit_n))['netN3','Std. Error'])
[1] 1.631465
```

We note that this interval lies entirely above the NIM. This means that we can say that N3 is non-inferior to N2, in terms of mosquito mortality.

7 Simulate trials to aid power calculations

Before carrying out an EHT, we should consider whether the trial is well-powered to answer the research question that is under consideration. Let's consider an example, where an EHT will be carried out to assess whether or not a novel ITN ('E2') is superior to a standard pyrethroid-only product ('E1'). We can use simulation-based methods to generate an estimate of trial power. When we simulate trials, we can select the average mortality induced by the ITNs. Let's imagine that we expect the pyrethroid-only net to kill 30% of mosquitoes (this value could be estimated from previous studies in the same region, or a different region with a similar level of pyrethroid resistance in the wild mosquito population). We may be unsure what mortality to expect in our novel product, but we could pose the question: what would be the smallest improvement that would be worth measuring? If this is a difference of 10%, we set the mortality of the novel ITN to be 40%. The next step is, for the proposed trial design, is to simulate the trial and see if the new net is judged to be superior to the pyrethroid-only ITN. As the simulation will be random, it is necessary to simulate many trials (say 500 or 1000), and calculate the proportion of trials for which a verdict of superiority is reached. This will form our power estimate.

Our work builds on a paper by Johnston et al., published in *Methods in Ecology and Evolution* in 2015 (`doi:10.1111/2041-210X.12306`). In that article, the authors presented a tool (which is written as a function in R) which can simulate a range of GLMMs, including the logistic regression models considered here. We will utilise their R function `simm.glmm`, to simulate an EHT. But first we must specify the trial design in R. Here we describe a seven-arm trial, containing 7 nets: an untreated control ("C") and 6 ITNs. Following Johnston *et al.* we rotate the nets around the huts each week:

```
latsq <-
  rbind(
    c("C", "E1", "E2", "E3", "E4", "E5", "E6"),
    c("E6", "C", "E1", "E2", "E3", "E4", "E5"),
    c("E5", "E6", "C", "E1", "E2", "E3", "E4"),
    c("E4", "E5", "E6", "C", "E1", "E2", "E3"),
    c("E3", "E4", "E5", "E6", "C", "E1", "E2"),
    c("E2", "E3", "E4", "E5", "E6", "C", "E1"),
    c("E1", "E2", "E3", "E4", "E5", "E6", "C"))

colnames(latsq) <- paste("hut", 1:nrow(latsq), sep = "")
rownames(latsq) <- paste("week", 1:ncol(latsq), sep = "")
latsq
      hut1 hut2 hut3 hut4 hut5 hut6 hut7
week1 "C"  "E1" "E2" "E3" "E4" "E5" "E6"
week2 "E6" "C"  "E1" "E2" "E3" "E4" "E5"
week3 "E5" "E6" "C"  "E1" "E2" "E3" "E4"
week4 "E4" "E5" "E6" "C"  "E1" "E2" "E3"
week5 "E3" "E4" "E5" "E6" "C"  "E1" "E2"
week6 "E2" "E3" "E4" "E5" "E6" "C"  "E1"
week7 "E1" "E2" "E3" "E4" "E5" "E6" "C"
```

Now we make a data frame, in which we'll store the data:

```
mosdata <-
  expand.grid(
  hut = factor(1:ncol(latsq)),
  week = factor(1:nrow(latsq)),
  night = factor(1:7)
  )
mosdata <- mosdata[order(mosdata$hut, mosdata$week, mosdata$night),]

mosdata$net <- factor(diag(latsq[mosdata$week, mosdata$hut]))
```

And we also need to specify where the volunteers will sleep each night.

```
aux <- rep(0,49) #enough data pts for a week
for(k in 1:7){ #Move the volunteers each night
  aux[(7*(k-1)+1):(7*(k-1)+7)] <- c( k : 7 , seq_len(k-1)  )
}
#repeat for all 7 weeks
mosdata$sleeper <- factor(rep(aux,7))
#Summarise data
table(mosdata$hut)
table(mosdata$net)
table(mosdata$sleeper)
```

Run the command `View(mosdata)` to take a look at the full dataset. We now add an identifier for each observation. We'll also define a variable `n`, for the number of mosquitoes entering each hut each night:

```
mosdata$observation <- factor(formatC(1:nrow(mosdata), flag="0", width=3))
mosdata$n <- 25 #Could also be random e.g.
#mosdata$n <- rnbinom(dim(mosdata)[1], mu = 10, size = 2)
```

Now we have the data frame needed to simulate a trial using the `sim.glmm` function. The mortality in the reference category should be entered on the log-odds scale (we can use the built-in function `qlogis` for this). The mortalities in the other arms should be entered as odd ratios. The random-effects should be described in terms of their variance (not standard deviation). Here we show an example of using the function on our data frame `mosdata`. We start by defining some odds ratios. Only E1 and E2 are involved in the power analysis, so it doesn't matter what we put for the other ITNs

```
OR1 <- (0.3/(1-0.3))/(0.05/(1-0.05))
OR2 <- (0.4/(1-0.4))/(0.05/(1-0.05))

mosdata <-
  sim.glmm(design.data = mosdata,
           fixed.eff = list(
             intercept = qlogis(0.05),
```

```
                net = log(c(C = 1, E1 = OR1, E2 = OR2, E3 = OR2,
                E4 = OR2, E5 = OR2, E6 = OR2))),
              rand.V = c(hut = 0.3, sleeper = 0.3, observation = 0.8),
              distribution = "binomial")
```

If we look again at our data frame, we can see a new variable called `response` has been added. This shows how many mosquitoes have been killed each night.

```
> head(mosdata)
    hut week night net sleeper  n observation response
1     1    1     1   C       1 25         001        1
50    1    1     2   C       2 25         002        6
99    1    1     3   C       3 25         003        5
148   1    1     4   C       4 25         004        0
197   1    1     5   C       5 25         005        2
246   1    1     6   C       6 25         006        0
```

Now, we can write a function to simulate a trial, and then make the superiority assessment. The function will return a value of 1 if superiority is found and a value of 0 if it isn't.

```
sim_sup <- function(...){
  #Enter fixed effects as odds ratios (compared to the control [intercept]).
  #Write the intercept on the log-odds scale
  OR1 <- (0.3/(1-0.3))/(0.05/(1-0.05))
  OR2 <- (0.4/(1-0.4))/(0.05/(1-0.05))

  mosdata <-
    sim.glmm(design.data = mosdata,
              fixed.eff = list(
                intercept = qlogis(0.05),
                net = log(c(C = 1, E1 = OR1, E2 = OR2, E3 = OR2,
                E4 = OR2, E5 = OR2, E6 = OR2))),
              rand.V = c(hut = 0.3, sleeper = 0.3, observation = 0.8),
              distribution = "binomial")

  #For the superiority test, should make E1 the intercept net
  mosdata2 <- mosdata
  #relevel
  mosdata2$net <- relevel(mosdata2$net,"E1")

  fit_model <-
    glmer(
      cbind(response, n - response) ~
        net + (1 | hut) + (1 | sleeper) + (1 | observation),
      family = binomial, data = mosdata2)

  if(coef(summary(fit_model))["netE2", "Pr(>|z|)"] <0.05 &
```

```
  coef(summary(fit_model))["netE2","Estimate"]>0){
    1
  }else{
    0
  }
}
```

The function can be run using `sim_sup()`. However, as the simulation process is random, a large number of trials should be simulated. The trial power is then the proportion of trials in which superiority is demonstrated. Here we show how to simulate 100 trials:

```
nsim <- 100
simulations <- sapply(1:nsim, sim_sup)
print(paste0('Power Estimate: ',100*sum(simulations)/length(simulations),'%'))
```

How many trials should be simulated will depend on the level of precision required for the power estimate. This can be found from the confidence intervals of the power estimate:

```
binom.test(table(factor(simulations,c(1,0))))$conf.int
[1] 0.5792331 0.7697801
```

This confidence interval is quite wide, indicating that more than 100 simulations should be used. For example, you could try re-running for 1000 simulations, and then check the width of the confidence intervals.

In the github repository(`https://github.com/JDChallenger/EHT_Visualise`), there is a file called `power_calculator_user_script.R`. This can be used to generate a power estimate for an experimental hut trial, as specified by the user. Separate functions are provided for evaluation of trials for ITNs & IRS (indoor residual spraying).
